# Angiotensin II-mediated MYH9 downregulation causes structural and functional podocyte injury in diabetic kidney disease

**DOI:** 10.1038/s41598-019-44194-3

**Published:** 2019-05-22

**Authors:** Jeong Suk Kang, Seung Joo Lee, Ji-Hye Lee, Ji-Hee Kim, Seung Seob Son, Seung-Kuy Cha, Eun Soo Lee, Choon Hee Chung, Eun Young Lee

**Affiliations:** 1 Department of Internal Medicine, Soonchunhyang University Cheonan Hospital, Cheonan, Korea; 2 Institute of Tissue Regeneration, College of Medicine, Soonchunhyang University, Cheonan, Korea; 3 Department of Pathology, Soonchunhyang University Cheonan Hospital, Cheonan, Korea; 4 Department of Physiology, Yonsei University Wonju College of Medicine, Wonju, Korea; 5 Department of Internal Medicine, Yonsei University Wonju College of Medicine, Wonju, Korea

**Keywords:** Molecular biology, Diabetic nephropathy

## Abstract

MYH9, a widely expressed gene encoding nonmuscle myosin heavy chain, is also expressed in podocytes and is associated with glomerular pathophysiology. However, the mechanisms underlying MYH9-related glomerular diseases associated with proteinuria are poorly understood. Therefore, we investigated the role and mechanism of MYH9 in diabetic kidney injury. MYH9 expression was decreased in glomeruli from diabetic patients and animals and in podocytes treated with Ang II *in vitro*. Ang II treatment and siRNA-mediated MYH9 knockdown in podocytes resulted in actin cytoskeleton reorganization, reduced cell adhesion, actin-associated protein downregulation, and increased albumin permeability. Ang II treatment increased NOX4 expression and ROS generation. The Ang II receptor blocker losartan and the ROS scavenger NAC restored MYH9 expression in Ang II-treated podocytes, attenuated disrupted actin cytoskeleton and decreased albumin permeability. Furthermore, MYH9 overexpression in podocytes restored the effects of Ang II on the actin cytoskeleton and actin-associated proteins. Ang II-mediated TRPC6 activation reduced MYH9 expression. These results suggest that Ang II-mediated MYH9 depletion in diabetic nephropathy may increase filtration barrier permeability by inducing structural and functional podocyte injury through TRPC6-mediated Ca^2+^ influx by NOX4-mediated ROS generation. These findings reveal a novel MYH9 function in maintaining urinary filtration barrier integrity. MYH9 may be a potential target for treating diabetic nephropathy.

## Introduction

Diabetic nephropathy is the major cause of end-stage renal disease (ESRD), which affects 20 to 40% of people with diabetes and is clinically characterized by proteinuria^1^. Podocyte damage and loss may be important in the pathogenesis of diabetic nephropathy. Podocytes are highly specialized and differentiated epithelial cells that maintain the structure and function of the glomerular capillary filtration barrier^2^. The complex structure podocytes depends on a highly structured actin cytoskeleton. Numerous studies have shown that glomerular kidney diseases causing various degrees of proteinuria are typically associated with foot process effacement and/or slit diaphragm disruption. Podocyte foot process effacement is often driven by the active reorganization of actin filaments^3,4^. Proteins regulating the plasticity of the podocyte actin cytoskeleton are vital for the maintenance of glomerular filter function^5–9^.

Nonmuscle myosin II (NM II) is an important motor protein that has actin cross-linking and contractile properties in many tissues, including podocytes. There are three isoforms of NM II heavy chains, MYH9, MYH10 and MYH14^10–12^. A subset of MYH9 and MYH10 messenger RNAs are expressed in human glomerular podocytes, whereas MYH14 is not expressed in glomrulus^12,13^. Many functional studies have shown that MYH9 is localized in the cytoplasm of mesangial cells and podocytes and that MYH10 is localized in mesangial cells, suggesting that these two myosin isoforms play either redundant or distinct biological roles depending on the cell type^12,14,15^.

Many studies have implicated MYH9 in glomerular pathophysiology. MYH9 expression is associated with alterations in the actin cytoskeleton, cell shape and adhesion properties^16^. Clinical studies have revealed associations of MYH9 with human immunodeficiency virus-related glomerulosclerosis, nondiabetic ESRD, and hypertensive nephrosclerosis in African-Americans and Hispanic-Americans^17–20^. Recently, the association of MYH9 was also described in diabetic ESRD patients^21,22^. In our previous study, MYH9 mutation was associated with proteinuria due to the foot process effacement of renal glomerular podocytes. The blockade of the renin-angiotensin system by losartan, an angiotensin receptor blocker, seems to improve proteinuria^23^. Although MYH9 is associated with proteinuria in the aforementioned nephropathies, its role and the mechanisms responsible for MYH9-related glomerular disease remain unclear.

MYH9 interacts dynamically with F-actin to contract the cytoskeleton and statically to maintain membrane tension and cell shape^24^. Podocyte foot process effacement, the hallmark of podocyte damage and proteinuric kidney diseases, is accompanied by the altered expression of podocyte proteins and the reorganization of the actin cytoskeleton^4^. Angiotensin II (Ang II) has been suggested to play roles in the rearrangement of the cytoskeleton of podocytes^25^. Elevated Ang II in diabetes can transform a podocyte from a dynamically stable state to an adaptively migratory state^26^. Accordingly, Ang II-induced MYH9 dysfunction might disrupt the cytoskeleton to cause proteinuria in diabetic podocytes. In this paper, we investigated the role of MYH9 and its mechanism responsible for diabetic kidney injury. Our findings implicate MYH9 as an important component in the maintenance of the biological function of podocytes and suggest pathways that might be affected by MYH9 function in the pathogenesis of diabetic nephropathy.

## Results

### Glomerular MYH9 expression is decreased in diabetic nephropathy

**Figure 1:**
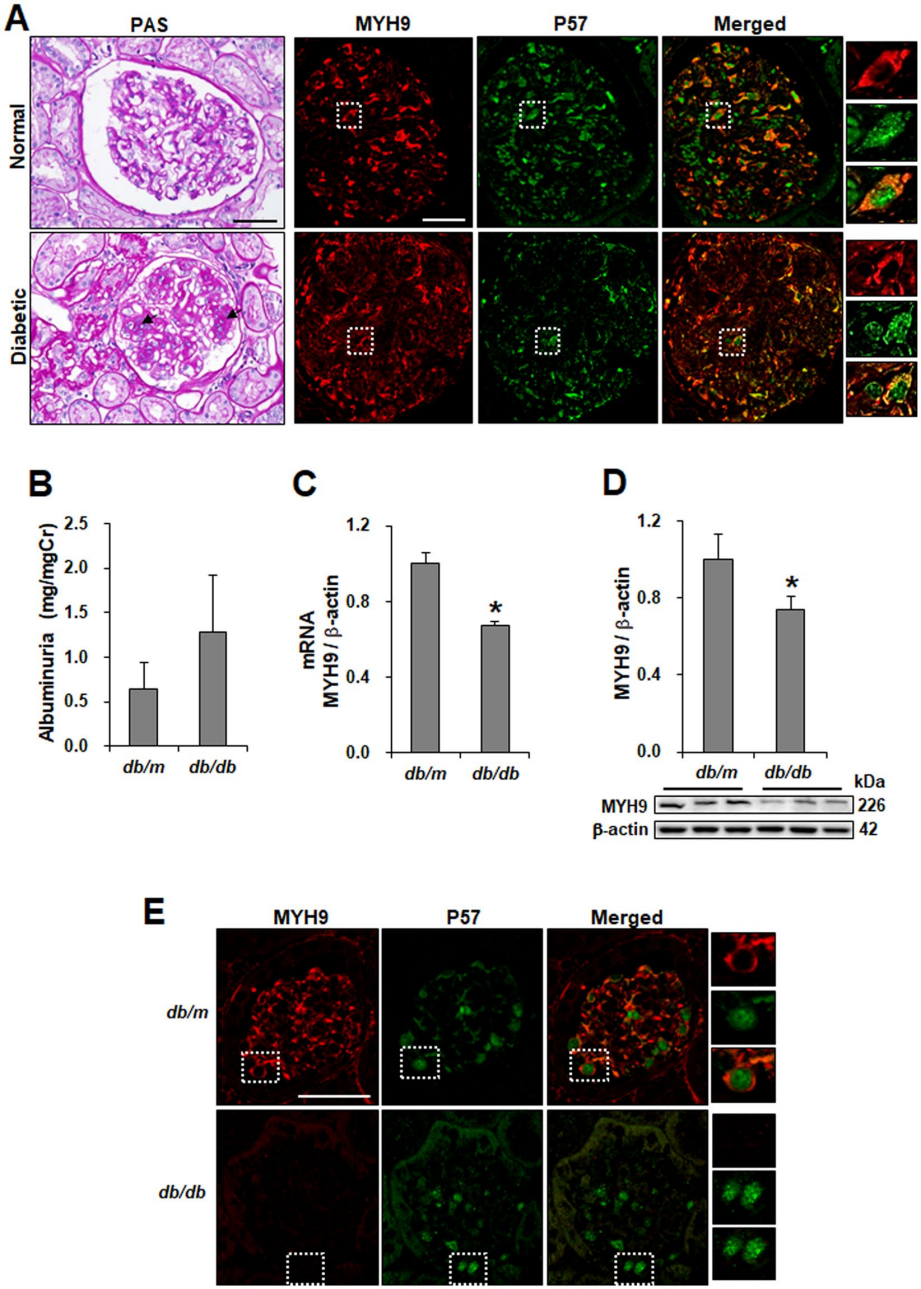
MYH9 is downregulated in the glomeruli of diabetic human and mouse kidney. (**A**) Representative sections of kidneys from paraffin-embedded normal and proteinuric diabetic human biopsies (*n* = 15) demonstrating the increased mesangial matrix (arrow) using PAS staining (left) and the reduction of MYH9 expression (red) using indirect IF staining in diabetic glomeruli (right). Podocyte-specific staining was performed with P57 (green). Magnification 20x; bar = 50 μm. (**B**) Increased albuminuria (mg/mg Cr, *n* = 9 per group) levels at 16 weeks of age in diabetic mice. (**C**) Real-time PCR and (**D**) Western blot demonstrating decreased MYH9 in diabetic *db/db* mice (*n* = 6) compared to nondiabetic *db/m* mice (*n* = 5). (**E**) Indirect IF staining with the MYH9 antibody in paraffin-embedded diabetic mouse kidneys. Magnification 40x; bar = 50 μm. Dashed white boxes are enlarged images of representative podocytes. Data are presented as the means ± SEM. *P < 0.05 versus nondiabetic *db/m* mice.

To investigate the loss of MYH9 in diabetic nephropathy, paraffin-embedded human kidney sections were double labeled with MYH9 and P57, a podocyte-specific marker, using immunofluorescence. In nondiabetic human glomeruli, several glomerular cells, including podocytes and mesangial cells, were stained positively for MYH9 (Fig. 1A, right). However, the expression of MYH9 was decreased in the glomeruli of diabetic patients, showing heavy proteinuria (Supplementary Table 1) and mesangial matrix expansion (Fig. 1A, left). In *db/db* diabetic mice with albuminuria, the renal expression of MYH9 was significantly decreased at the mRNA and protein levels compared to that in the control (Fig. 1B–D). Similar to the findings in human diabetic kidneys, MYH9 detected in the cytoplasm of podocytes was decreased in diabetic podocytes (Fig. 1E). These findings were also consistently observed in the kidneys of diabetic Otsuka Long-Evans Tokushima Fatty (OLETF) rats (Supplementary Fig. S1A,B).

### Decreased expression of MYH9 by Ang II treatment in cultured podocytes

**Figure 2:**
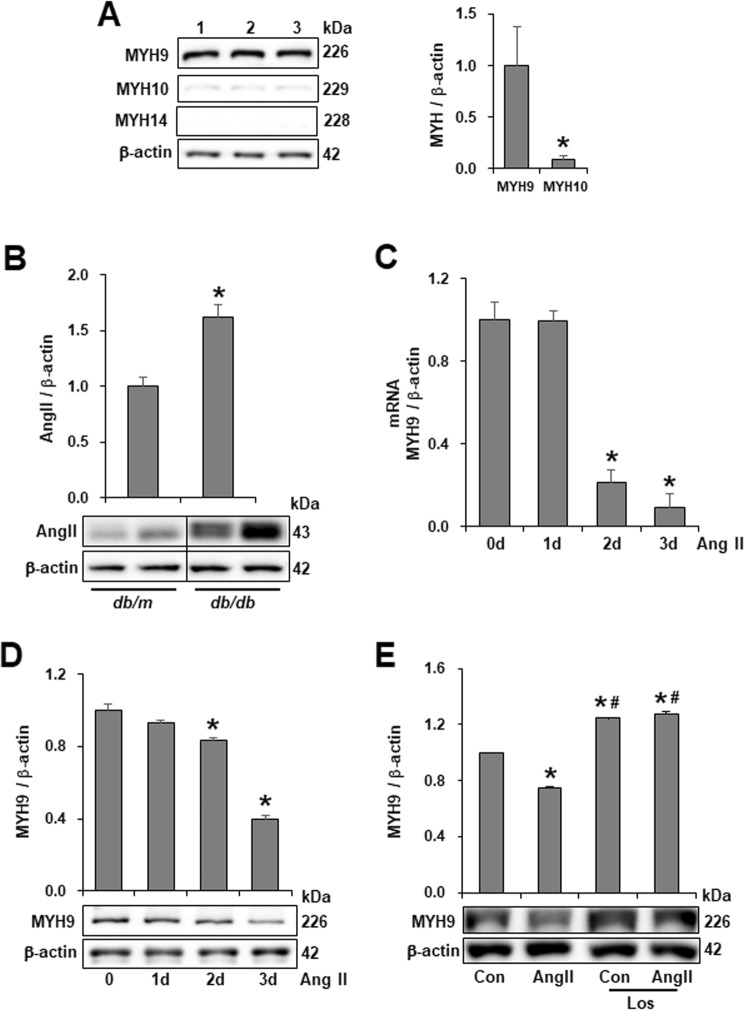
Downregulation of MYH9 protein in Ang II-treated podocytes. (**A**) Lysates from cultured mouse podocytes were Western blotted with antibodies, specific to MYH9, MYH10 and MYH14 to determine their expression levels (*n* = 3). (**B**) Western blot demonstrating increased Ang II in diabetic *db/db* mice (*n* = 8) compared to nondiabetic *db/m* mice (*n* = 4). The blots were cropped from different parts of the same gel. (**C**) Real-time PCR and (**D**) Western blot demonstrating decreased MYH9 at the RNA and protein levels in Ang II (10^−6^ M)-stimulated podocytes in a time-dependent manner (*n* = 3). (**E**) Western blot of MYH9 in mouse podocytes stimulated by Ang II with or without treatment with losartan (Los, 10^−6^ M, *n* = 3 per group). Data are presented as the means ± SEM. Similar results were obtained in two independent experiments. *P < 0.05 versus MYH9, nondiabetic *db/m* mice and control podocytes. ^#^P < 0.05 compared with podocytes treated with Ang II.

To analyze the expression of three isoforms of NM II in glomeruli and podocytes, mouse kidney tissue and cell lysates from cultured mouse podocytes were subjected to immunofluorescence and Western blotting. Unlike MYH9 expressed in podocytes and mesangial cells, MYH10 was observed only in mesangial cells and MYH14 was not detected in glomeruli (Supplementary Fig. S1C). In differentiated podocytes, MYH9 protein was prominently detected, whereas MYH10 and MYH14 were weakly observed (Fig. 2A). Differentiated podocytes showed MYH9 immunoreactivity along the entire length of stress fibers, and merged images of MYH9 with F-actin and synaptopodin showed their colocalization in podocytes (Supplementary Fig. S2A). Compared to undifferentiated cells, differentiated podocytes expressed increased MYH9 at the mRNA and protein levels (Supplementary Fig. S2B,C). These data show that MYH9 is required to maintain the biological function of the podocyte cytoskeleton.

Ang II, a key contributor to the induction of diabetic glomerular disease, was upregulated in the kidneys of diabetic *db/db* mice (Fig. 2B) and exhibited a time-dependent effect on the loss of MYH9 at the RNA and protein levels (Fig. 2C,D). The Ang II-induced MYH9 reduction was restored by the Ang II type 1 receptor antagonist losartan (Fig. 2E).

### Podocyte actin cytoskeleton integrity is MYH9 dependent

**Figure 3:**
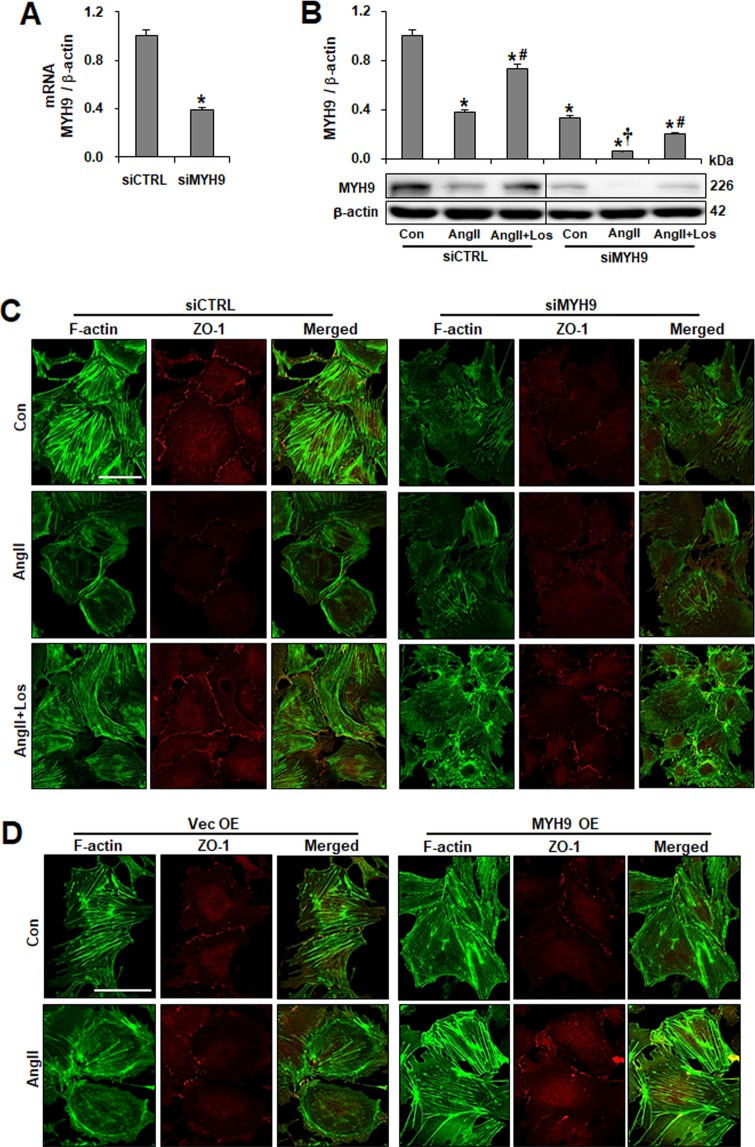
Effect of downregulated MYH9 expression on the F-actin network. (**A**) Validation of siRNA knockdown of *Myh9* (siMYH9) in podocytes using real-time PCR, corrected by β-actin mRNA levels in the same sample. (**B**) Western blots of MYH9 after siRNA-mediated inhibition of MYH9 in control (Con) and Ang II-treated podocytes with or without losartan (Los). The blots were cropped from different parts of the same gel. (**C**) Cultured podocytes grown on coverslips were fixed with 4% PFA and immunolabeled with FITC-phalloidin (green) and junctional marker ZO-1 (red). Control cells display uniformed actin stress fibers and ZO-1. Treatment of podocytes with Ang II resulted in actin rearrangement and loss of ZO-1 staining. MYH9-depleted cells showed disorganized, shortened and decreased stress fibers. Treatment of Ang II-stimulated control or MYH9-depleted cells with losartan restored actin stress fibers and ZO-1 staining. (**D**) podocytes transfected with GFP-MYH9 were analyzed by immunofluorescence with FITC-phalloidin and ZO-1 antibodies. Magnification 40x; bar = 50 μm. Data are presented as the means ± SD, *n* = 3. Similar results were obtained in three independent experiments. *P < 0.05 compared with the siCTRL. ^#^P < 0.05 compared with siCTRL and siMYH9 control podocytes treated with Ang II. ^†^P < 0.05 compared with the siMYH9 control. Abbreviations: siCTRL, scrambled control; siMYH9, MYH9 siRNA transfection.

To investigate the effect of the MYH9 reduction on actin networks, MYH9 siRNA transfection was performed to knockdown MYH9 expression *in vitro*. Compared to control cells, the transfection of podocytes with MYH9 siRNA resulted in 60% diminished MYH9 expression at both the mRNA and protein levels (Fig. 3A,B). The transfected podocytes exhibited a greater loss (approximately 90%) of MYH9 expression than did control cells in the presence of Ang II. The Ang II-induced reduction of MYH9 was restored by losartan. The knockdown of MYH9 was further analyzed by immunofluorescence using an anti-MYH9 antibody, confirming the reduction of MYH9 in MYH9 siRNA-transfected podocytes (Supplementary Fig. S3A). To assess whether MYH9 suppression could lead to cytoskeleton rearrangement and changes in cell-cell junctions, podocytes were visualized by double staining with FITC-phalloidin dye and the junctional marker ZO-1 (Fig. 3C). Control cells exhibited uniformed actin stress fibers throughout the cytoplasm. Ang II induced disorganized, cortically rearranged and decreased actin stress fibers, and these changes were reversed by losartan. MYH9 knockdown podocytes showed disorganized, shortened and decreased actin stress fibers. Compared with unstimulated conditions, after stimulation with Ang II, there were no detectable changes in MYH9-depleted podocytes. The expression of ZO-1 at the cell junction was reduced in Ang II-treated and MYH9 knockdown podocytes compared to that in control siCTRL podocytes. Similar to F-actin, losartan treatment recovered the ZO-1 levels. To investigate the effect of overexpressed MYH9, podocytes were transfected with mouse GFP-MYH9 cDNA or control cDNA. The expression of synaptopodin was reduced by Ang II-treated and MYH9 knockdown podocytes, as shown in Supplementary Fig. S3B. However, MYH9-overexpressing podocytes showed nonreduced synaptopodin expression and uniformed actin stress fiber under Ang II treatment (Supplementary Fig. S3C,D). Furthermore, the reduced expression of ZO-1 at the cell junction by Ang II was not observed in MYH9-overexpressing cells (Fig. 3D). These data suggest that exogenously treated MYH9 could restore the effects of Ang II on actin cytoskeleton and podocyte functions.

### MYH9 depletion decreases podocyte adhesion and actin-binding proteins

**Figure 4:**
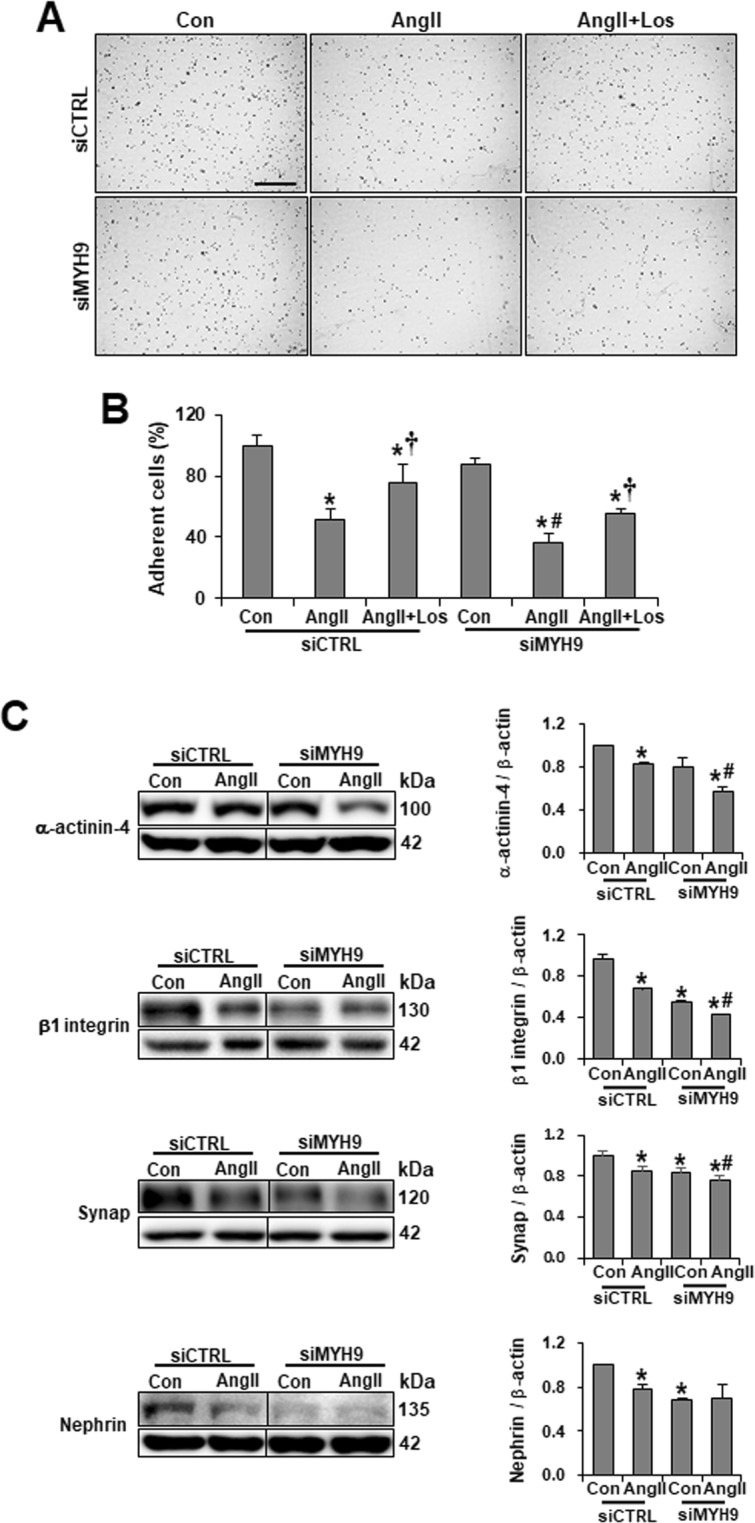
Loss of MYH9 affects podocyte attachment and the expression of actin-associated proteins. (**A**) Cell adhesion assays were performed by seeding Ang II-treated or MYH9 siRNA transfected podocytes onto the culture dish for 1 h (*n* = 5 per group). Adhered cells were fixed and stained with crystal violet. Magnification 10x; bar = 300 μm. (**B**) Quantification of results in panel A. (C) Western blots demonstrating decreased α-actinin-4, β1 integrin, synaptopodin and nephrin in MYH9-depleted podocytes. The blots were cropped from different parts of the same gel. Data are presented as the means ± SD, *n* = 3. Similar results were obtained in two independent experiments. *P < 0.05 compared with the siCTRL control. ^#^P < 0.05 compared with the siMYH9 control. ^†^P < 0.05 compared with respective Ang II-treated podocytes. Abbreviations: Synap, Synaptopodin.

An adhesion assay was performed to investigate the effect of MYH9 on podocyte anchorage to the glomerular basement membrane. Podocyte attachment to collagen I-coated dishes showed that Ang II reduced the adherence of podocytes. Decreased adherence was restored by treatment with losartan. MYH9-depleted podocytes were less adherent than the control cells, and in response to Ang II stimulation, these cells exhibited markedly reduced podocyte adhesion (Fig. 4A,B). To investigate whether the decreased podocyte adherence was mediated by the altered expression of actin-associated proteins and cell adhesion complexes, the expression levels of α-actinin-4 (actin cross-linker protein), β1 integrin (a cell-matrix adhesion receptor), synaptopodin, and nephrin (slit diaphragm protein) were examined (Fig. 4C). Ang II treatment exhibited the decreased expression of these proteins. MYH9-depleted podocytes also exhibited a loss of protein expression, while Ang II stimulation induced an even further reduction in the expression of these proteins. These results suggest that reduced MYH9 expression may be associated with the expression of the actin-associated proteins localized at the podocyte filtration barrier or slit diaphragm.

### Association of MYH9 with NOX4-mediated ROS generation evoked by Ang II

**Figure 5:**
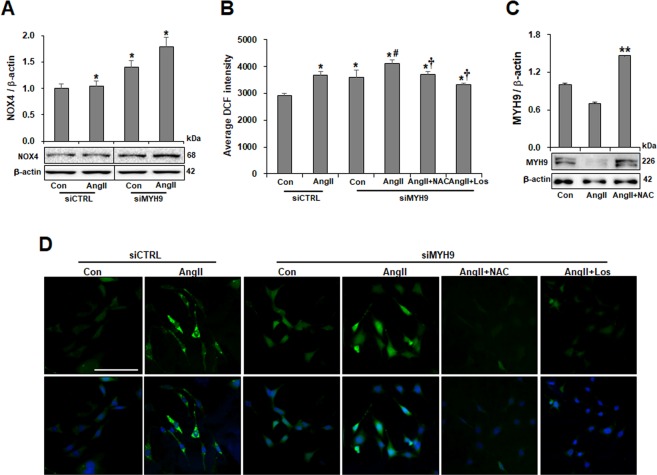
Association of MYH9 expression with NOX4-mediated ROS. (**A**) Western blot demonstrating increased NOX4 in podocytes (*n* = 3). The blots were cropped from different parts of the same gel. (**B**) Histogram analysis of the results shown in (**D**). For the comparison of 2′,7′-dichlorofluorescein (DCFDA) intensities, ROS generation was measured among different groups using a fluorometer II (*n* = 4 per group). (**C**) Western blot demonstrating the restored expression of MYH9 by NAC. (**D**) Representative images of ROS measurements in podocytes loaded with DCFDA (top) and merged images with DAPI and DCFDA (bottom) to show cell number. Data are presented as the means ± SD. The experiments were repeated twice. Magnification 20x; bar = 300 μm. *P < 0.05 compared with the siCTRL control. ^#^P < 0.05 compared with the siMYH9 control. ^†^P < 0.05 compared with siMYH9 control podocytes treated with Ang II. **P < 0.05 compared to control.

Ang II-induced NOX4 leads to the generation of ROS^27–30^. To investigate the effect of the association of Ang II-mediated NOX4 protein synthesis with ROS generation on MYH9 expression, NOX4 expression and ROS generation were investigated. Ang II-treated podocytes showed increased NOX4 expression. MYH9-depleted podocytes displayed increased NOX4 production and NOX4 upregulation in response to Ang II (Fig. 5A). ROS generation was induced in podocytes stimulated by Ang II and recovered by losartan and the ROS scavenger N-acetyl-L-cysteine (NAC) (Supplementary Fig. S4A,B). Furthermore, MYH9-depleted podocytes displayed greater basal ROS production than did scrambled control transfected cells and had significantly enhanced ROS generation upon Ang II stimulation. The increased production of ROS was attenuated by both NAC and losartan treatments (Fig. 5B,D). The Ang II-induced reduction of MYH9 expression was significantly restored by NAC treatment (Fig. 5C). These results suggest that the NOX4-mediated ROS generation evoked by Ang II mediates MYH9 expression.

### Blocking ROS generation attenuates MYH9 depletion-induced actin cytoskeleton disruption and filtration barrier dysfunction in podocytes

**Figure 6:**
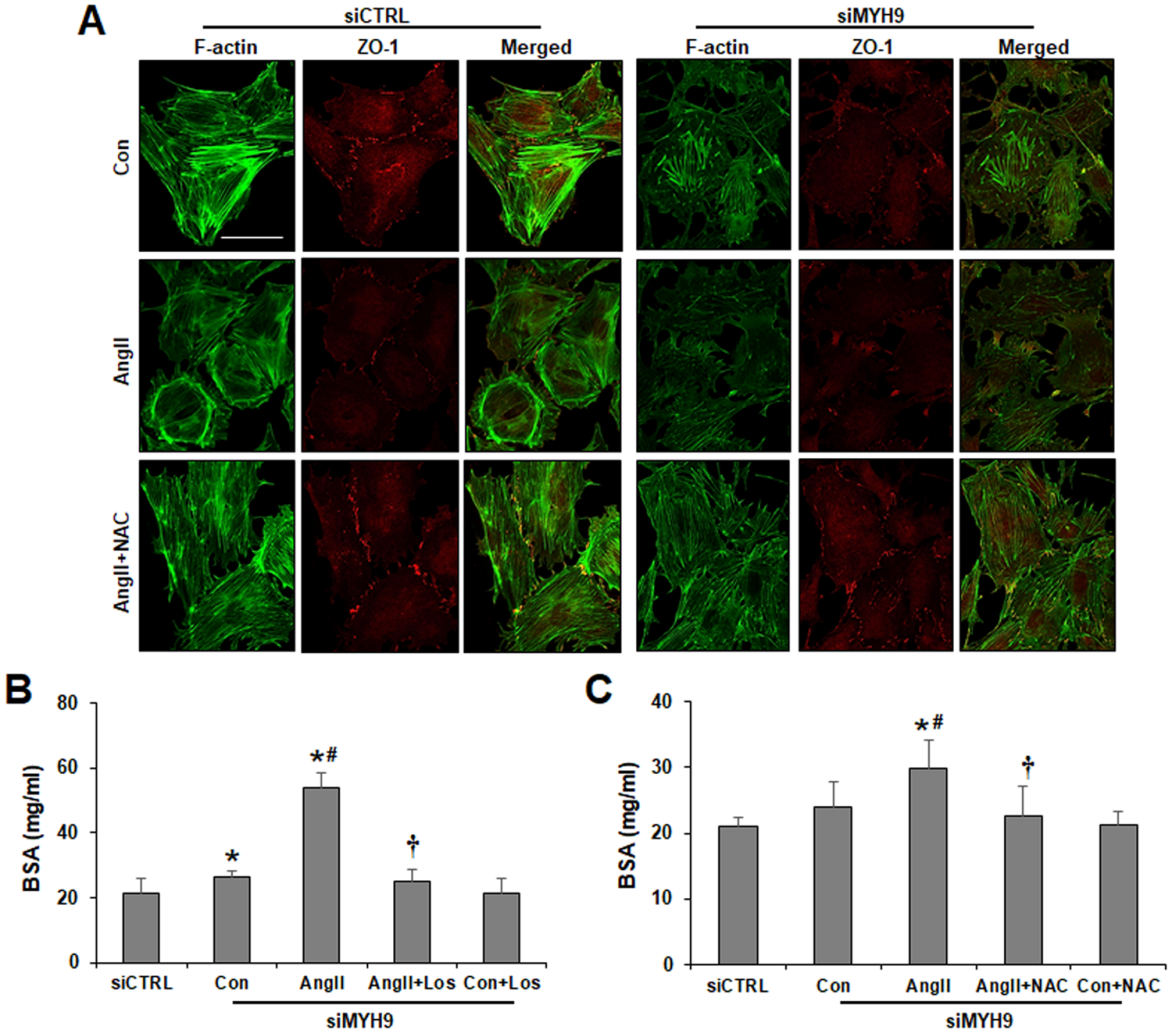
Attenuation of MYH9 depletion-induced actin cytoskeleton disruption and impaired filtration barrier by NAC. (**A**) Cultured podocytes grown on coverslips were fixed with 4% PFA and immunolabeled with FITC-phalloidin (green) and junctional marker ZO-1 (red). NAC-treated control or MYH9-depleted podocytes showed restored actin stress fibers and ZO-1 staining. Magnification 40x; bar = 50 μm. (**B**) Losartan and (**C**) NAC effect on podocyte permeability. Podocytes on the Transwell filter chamber were treated with Ang II in the presence of losartan or NAC. Then, 40 mg/ml BSA-containing medium was added into the lower chambers, and the upper chambers were sampled. The data are presented as the means ± SD, *n* = 3. The experiments were repeated twice. *P < 0.05 compared with siCTRL. ^#^P < 0.05 compared with the siMYH9 control. ^†^P < 0.05 compared with siMYH9 control podocytes treated with Ang II.

To assess whether ROS suppression could rescue MYH9 depletion-induced actin filament reorganization and change cell-cell junctions, NAC-treated podocytes were subjected to immunofluorescence. Similar to losartan stimulation, as shown in Fig. 3C, Ang II- or siMYH9-induced actin cytoskeleton rearrangement and ZO-1 reduction were restored by NAC treatment (Fig. 6A). Next, a permeability assay was performed to investigate the effect of MYH9 on podocyte filtration barrier function. Increased albumin permeability was observed in MYH9 knockdown podocytes under Ang II treatment, whereas NAC and losartan treatment prevented albumin permeability (Fig. 6B,C). These results suggest that the restored expression of MYH9 by NAC and losartan ameliorates podocyte actin cytoskeleton and filtration barrier function.

### Involvement of TRPC6 in Ang II-induced Ca2^+^ influx and MYH9 downregulation

**Figure 7:**
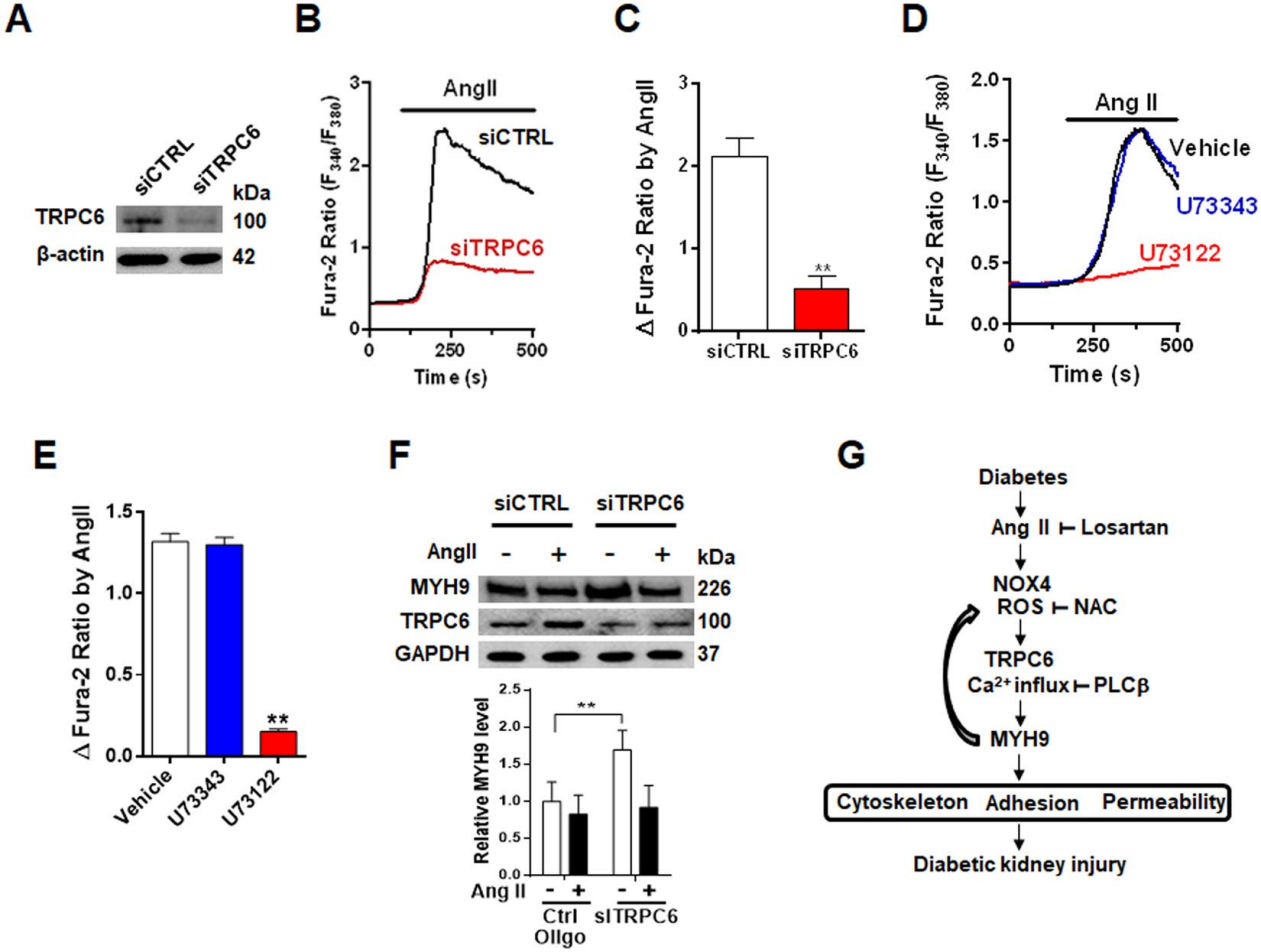
Upregulation of MYH9 expression in TRPC6-depleted podocytes. (**A**) Validation of siRNA knockdown of *Trpc6* (siTRPC6) in podocytes using Western blot. (**B**) Representative traces showing that Ang II-activated [Ca2^+^]_i_ was inhibited in TRPC6-depleted podocytes compared to control cells (siCTRL). (**C**) Summary of the results in panel B. (**D**) Representative traces showing that Ang II (1 μM, 1 h)-induced [Ca2^+^]_i_ increase was blunted by U73122, the active form of a PLCβ inhibitor, but not by its inactive analog (U73343, 2.5 μM, 1 h). (**E**) Summary of the results in panel E. (**F**) Western blot demonstrating the effects of siRNA knockdown of *Trpc6* (siTRPC6) on MYH9 upregulation. (**G**) Schematic representation of the possible signaling pathway leading to podocyte injury by Ang II-mediated MYH9 downregulation. **P < 0.01.

Transient receptor potential canonical channel 6 (TRPC6) is a downstream target of Ang II receptor signaling, and altered TRPC6 expression and Ca^2+^ influx have been associated with Ang II-induced podocyte injury^31–33^. Consistent with a previous study, *Trpc6* knockdown in podocytes by siRNA significantly inhibited Ang II-induced Ca^2+^ influx compared to that in control cells (Fig. 7A–C). Gαq-coupled Ang II receptor activates phospholipase C-beta (PLCβ) to produce a second messenger, including diacylglycerol, to activate TRPC6 directly^34^. Ang II-mediated Ca^2+^ influx was blunted by pretreatment with the PLCβ inhibitor U73122 (Fig. 7D,E). Next, we examined whether Ang II-stimulated TRPC6-mediated Ca^2+^ influx regulates MYH9 expression. While Ang II treatment increased TRPC6 expression, MYH9 expression was downregulated by Ang II (Fig. 7F). Moreover, *Trpc6* knockdown podocytes significantly enhanced MYH9 expression. These data suggest that TRPC6 is the primary Ca^2+^ influx mechanism linking to MYH9 regulation in podocytes.

## Discussion

This study provides evidence that MYH9 downregulation in diabetic nephropathy induces podocyte dysfunction through the reorganization of the actin cytoskeleton, which is driven by TRPC6-mediated Ca^2+^ influx by NOX4-mediated ROS generation. MYH9 expression was decreased in podocytes from diabetic humans, mice, and rats. In cultured podocytes, the downregulation of MYH9 using Ang II and MYH9 siRNA resulted in actin cytoskeleton reorganization, reduced cell adhesion, and actin-associated protein downregulation at the podocyte filtration barrier or slit diaphragm. Podocytes treated with Ang II and MYH9 siRNA displayed increased NOX4 expression, ROS generation, and glomerular filtration barrier permeability. These changes were exacerbated in MYH9 knockdown podocytes in the presence of Ang II. Losartan and ROS inhibitor, NAC attenuated the disruption of the actin cytoskeleton, albumin permeability induced by MYH9 depletion, as repaired MYH9 expression. Furthermore, overexpressed MYH9 in podocytes could restore the effects of Ang II on the actin cytoskeleton and actin-associated proteins. The Ang II-enhanced TRPC6 expression by PLCβ and TRPC6-mediated Ca^2+^ influx are associated with a loss of MYH9 expression.

The MYH9 gene has been associated with many proteinuric kidney diseases^17,21^. We reported that MYH9-related nephropathy due to heterozygous MYH9 mutation manifests as global sclerosis, mild tubular atrophy, interstitial fibrosis, and proteinuria because of the foot process effacement of renal podocytes. The blockade of the renin-angiotensin system by angiotensin receptor blocker treatment improved proteinuria^23^. Angiotensin-converting enzyme (ACE) inhibitor induces the phosphorylation of MYH9, which suggests that MYH9 might contribute to the ACE signaling cascade^35^. The present study is the first to show the loss of MYH9 expression in the podocytes of diabetic human glomeruli and that Ang II receptor type 1 antagonists can improve the downregulated MYH9 expression and function in cultured podocytes.

*MYH9* encodes NM IIA and is expressed in many tissues, including glomerular podocytes^36^. MYH9 is a motor protein that binds to actin to regulate cellular motility. Tobias *et al*. revealed the essential role of MYH9 in the formation of the glomerular tuft and the filtration barrier^37^. Yingfan *et al*. reported that podocyte-ablated MYH9 mice showed podocyte abnormalities, albuminuria, and progressive glomerulosclerosis^38^. In contrast, Duncan *et al*. reported that the podocyte-specific deletion of *MYH9* in C57BL/6 mice background had no overt phenotype but was sufficient to induce glomerulosclerosis in response to a second provocation^39^. In addition to identifying the genetic loci responsible for strain-sensitive glomerulosclerosis, Duncan *et al*. generated the same deletion of *MYH9* on a mixed background and found no evidence of glomerulosclerosis, leading the authors to propose that *MYH9* is not absolutely required in adult podocytes^40^. Despite extensive studies by several groups, it remains contentious whether the loss of MYH9 is an early event in the disarrangement of the podocyte cytoskeleton or is a secondary disturbance due to some other provocations. Our findings on the basis of MYH9-depleted podocytes suggest that the loss of MYH9 is involved in the progression of podocyte dysfunction under diabetic disease-associated conditions.

MYH9 localizes to the podocyte foot process and is responsible for the movement of actin filaments and directly and indirectly interacts with many other proteins within the cytoskeletal protein network to maintain the normal structure of the cell^41^. Mutations in proteins that are specific for podocyte development and function, including α-actinin-4, nephrin, podocin, CD2AP, and TRPC6, lead to renal disease that is caused by the rearrangement of the actin cytoskeleton and the disruption of the filtration barrier^6–9^. α-Actinin-4 is one of four isoforms of α-actinin and is present along with actin stress fibers in regions of focal adhesion. The protein binds to a number of adhesion molecules including dystroglycans and integrins, which link podocytes to the glomerular basement membrane by an intracellular actin cytoskeleton^42–44^. Continuous remodeling of the actin cytoskeleton regulates cell migration, changes in cell morphology, and adhesive properties^4^. Presently, our results demonstrated that the loss of MYH9 by Ang II and endogenous MYH9 knockdown was associated with the expression level of actin-binding proteins at the slit diaphragm. In a previous study, Arie *et al*. demonstrated that neprhin colocalized with the motor protein Myo1c and that its dynamic organization is regulated by Myo1c at the slit diaphragm^45^. Similar to that of Myo1c, the MYH9-dependent molecular mechanism might affect the dynamic interaction of actin-associated proteins at the podocyte filtration barrier or slit diaphragm and is important for podocyte function.

In diabetic nephropathy, many putative pathways might contribute to the downregulation of MYH9 expression. Oxidative stress is a crucial mediator of the development and progression of diabetic nephropathy^46^. ROS generation is increased in podocytes exposed to Ang II^27^. NOX4 is a major source of ROS in the kidney and is enhanced in Ang II-treated podocytes^28–30^. In this study, NOX4 expression was upregulated in Ang II-stimulated podocytes, and NOX4-derived ROS generation mediated the downregulation of MYH9 and the reorganization of the F-actin cytoskeleton. Furthermore, MYH9 siRNA significantly increased NOX4 expression and oxidative stress. In the presence of Ang II, oxidative stress was exacerbated by the upregulation of NOX4. Blocking ROS by NAC attenuated the MYH9-induced disruption of the podocyte actin cytoskeleton and the dysfunction of the filtration barrier.

The dysfunction of calcium signaling in podocytes results in cytoskeleton rearrangement, foot process effacement, and proteinuria^47^. TRPC6 is located on the podocyte membrane and is an important mediator of podocyte calcium handling and actin dynamics^48^. Ang II can elevate albuminuria by activating TRPC6 channels in podocytes^49^, and Ang II-induced podocyte injury is involved in altered TRPC6 expression and Ca^2+^ influx^50^. Diabetic kidney disease showed that NOX-derived ROS production promotes podocyte injury by enhancing Ca^2+^ influx *via* the TRPC6 channel^51^. Ca^2+^-dependent remodeling of the actin cytoskeleton is also regulated by the Rho family of small guanosine triphosphatases (Rho GTPases), and TRPC6-mediated Ca^2+^ influx increases RhoA activity^52,53^. Although further investigations are required to verify whether TRPC6-mediated Ca^2+^ influx by NOX4-derived ROS evoked by Ang II contributes to the downregulation of MYH9, in this study, we suggest that elevated Ang II in diabetes induces MYH9 downregulation through TRPC6 activation-mediated Ca^2+^ influx by NOX4-derived ROS. Reduced MYH9 expression alters the actin cytoskeleton and actin-associated proteins, resulting in the loss of filtration barrier function (Fig. 7G).

In conclusion, our study suggests that MYH9 is essential for maintaining podocyte actin cytoskeleton integrity and preserving the glomerular filtration barrier. The collective data also indicate that MYH9 function is disrupted in diabetic mellitus, which may contribute to the pathogenesis of diabetic kidney injury.

## Methods

### Podocyte culture and treatment

Conditionally immortalized mouse podocytes were kindly provided by Dr. Peter Mundel (Harvard Medical School, Boston, MA, USA) and were cultured as described previously^54^. Generally, podocytes were cultured at 33 °C under permissive conditions in DMEM supplemented with 10% FBS and 10 U/ml mouse recombinant interferon-γ (Sigma-Aldrich, St. Louis, MO, USA) to enhance the expression of a thermosensitive T antigen. To induce differentiation, podocytes were grown under nonpermissive conditions at 37 °C in the absence of interferon-γ for 14 days. Cell confluence was 70–80% prior to Ang II treatment. Before the application of Ang II, the cells were maintained under serum-deprived conditions for 24 h and harvested for the next assay.

### Human biopsies

The study was approved by the Institutional Reviewed Board (IRB) of Soonchunhyang University Cheonan Hospital, Cheonan, Korea. The IRB waived the need to obtain informed consent from participants. The methods in this study were performed in accordance with the relevant guidelines and regulations. We retrospectively analyzed the data of 15 patients who underwent renal biopsy for the diagnosis of proteinuric diabetic nephropathy from January 2012 to December 2012 (Supplementary Table 1). Medical records were reviewed for clinicopathological information. All cases were histopathologically re-examined by one pathologist (JH Lee) to confirm the diagnosis and pathological features of glomerular injury including glomerular basement membrane thickening, podocyte foot process effacement, and global sclerosis. Nondiabetic samples were acquired from a nephrectomy specimen obtained during surgery to treat renal cell carcinoma; the sample was free of neoplastic and glomerular lesions. Periodic acid-Schiff (PAS) staining was analyzed on microscopes (Leica, Wetzlar, Germany).

### Animals

All experiments were approved by the Institutional Animal Care and Use Committee of Yonsei University Wonju College of Medicine, Wonju, Korea, and the methods were performed in accordance with the relevant guidelines and regulations. Male diabetic *db/db* mice (C57BLKS/J- *Leprdb*/*Leprdb*, *n* = 10) and male nondiabetic *db/m* mice (C57BLKS/J- *Leprdb*/+, *n* = 10) were purchased at 6 to 7 weeks of age from Jackson Laboratory (Bar Harbor, MA, USA). Long-Evans Tokushima Otsuka (LETO, nondiabetic control model, *n* = 10) and OLETF (Type 2 diabetic mellitus model, *n* = 10) rats were purchased from Otsuka Pharmaceutical Co., Ltd. (Tokushima, Japan). The animals were anesthetized with Zoletil (Virvac Laboratories, Carros, France) and xylazine hydrochloride (Rompun TS, Bayer AG, Leverkusen, Germany) by intraperitoneal injection at 16 and 46 weeks of age, respectively.

### Assessment of albuminuria

Urine was collected over 18 h using a metabolic cage. Total urine volume was measured and albumin and creatinine levels were quantified using albumin (ExocellNephrat II; Exocell Inc., Philadelphia, PA, USA) and creatinine (The Creatinine Companion; Exocell Inc.) ELISA kits according to the manufacturer’s directions. The urinary albumin-to-creatinine ratio was calculated as the ratio of urine to creatinine, expressed in milligrams per milligrams.

### siRNA and plasmid DNA transfection

Nontargeting control oligonucleotides (sc37007), MYH9 siRNA (sc61121), and TRPC6 siRNA (sc42673) were purchased from Santa Cruz Biotechnology. Differentiated podocytes were transfected with 20 nM siRNA mixed with DharmaFECT 1 siRNA transfection reagent (Thermo Fisher Scientific Inc., Lafayette, CO, USA) per the manufacturer’s instructions. Gene knockdown was assessed at 48 h by determining mRNA levels using quantitative real-time PCR. For assessing protein levels, Western blotting was performed at 72 h after siRNA transfection. Functional assays for siRNA-mediated gene silencing were performed at 72 h after siRNA treatment. CMV-GFP-NMHC II-A was a gift from Robert Adelstein (Addgene plasmid #11347; http://n2t.net/addgene:11347; RRID:Addgene_11347)^55^. Transfection was carried out by using Lipofectamine® LTX Reagent (Thermo Fisher Scientific) according to the manufacturer’s instructions. Twenty-four hours after transfection, the transfected cells were processed for Ang II treatment.

### Western blotting

Cultured podocytes and kidneys were homogenized in PRO-PREP^TM^ protein extraction solution (iNtRON Biotechnology, Seoul, Korea) containing protease inhibitor cocktail (Roche Diagnostics GmbH, Mannheim, Germany). All samples were quantified using the Bradford assay (Bio-Rad, Hercules, CA, USA) with BSA as a standard, and an equal amount of each lysate was examined by SDS-PAGE. The separated proteins were transferred to a polyvinylidene fluoride membrane (Millipore, Billerica, MA, USA). The membrane was blocked with 5% nonfat dry milk followed by primary antibody incubation at 4 °C overnight. Primary antibodies for MYH9 (Proteintech, Rosemont, IL, USA), MYH10 (Proteintech), MYH14 (Proteintech), nephrin (Progen Biotechnik, Heidelberg, Germany), NADPH oxidase 4 (NOX4, Santa Cruz Biotechnology), α-actinin-4 (Santa Cruz Biotechnology, Santa Cruz, CA, USA), β1 integrin (Santa Cruz Biotechnology), and synaptopodin (Synaptic Systems, Gottingen, Germany) were prepared in 0.1% Tris-buffered saline containing Tween-20 and 1% milk at an appropriate dilution. Subsequently, the membranes were washed with phosphate-buffered saline-Tween solution followed by incubation with horseradish peroxidase-conjugated secondary antibody. The bands were visualized with a ChemiDoc^TM^ XRS+ (Bio-Rad) imaging system using a Luminata Forte enhanced chemiluminescence solution (Millipore).

### Immunofluorescence

Podocytes were grown on collagen-coated coverslips for 14 days, fixed with 4% paraformaldehyde (PFA), permeabilized with 0.25% Triton X-100, blocked with 1% BSA, incubated with primary antibodies for 1 h at room temperature, and finally incubated with secondary antibodies for 1 h at room temperature. Primary antibodies against the following proteins were used: ZO-1 (Invitrogen), syanptopodin (Synaptic Systems), MYH9 and MYH10 (a gift from Dr. Lawrence B. Holzman)^56^, MYH14 (Proteintech), ZO-1 (Invitrogen), FITC-phalloidin (Sigma-Aldrich), and Rhodamine-phalloidin (Invitrogen). The images were collected using an LSM 510 META laser-scanning confocal microscope (Carl Zeiss Microimaging, Thornwood, NY, USA).

### Quantitative real-time PCR

Total RNA was isolated from cultured podocytes using TRIzol (Sigma-Aldrich) according to the manufacturer’s instructions. First-strand cDNA was synthesized from 1 μg of total RNA using a ReverTraAce^®^ qPCR RT Master Mix (TOYOBO, Tokyo, Japan) according to the manufacturer’s protocol. The cDNA was then amplified using SYBR Green PCR Master Mix (TOYOBO) for real-time PCR. The following primer pairs were used to assay the expression of α-actinin-4, β1 integrin, and nephrin: α-actinin-4: 5′-actaccacgcagcgaacc-3′ (forward) and 5′-tcccctgaaatgacctcc-3′ (reverse); β1 integrin: 5′-gaggttcaatttgaaattag-3′ (forward) and 5′-ggctctgcactgaacacatt-3′ (reverse); and nephrin: 5′-gaggaggatcgaatcaggaa-3′ (forward) and 5′-ggtccacttctgctgtgcta-3′ (reverse). Real-time PCR was carried out on a CFX Connect^TM^ system (Bio-Rad), and the data were analyzed following the ΔΔC_T_ method.

### Measurement of cytosolic ROS generation

Cytosolic ROS generation was measured using 2′-7′ dichlorofluorescindiacetate (CM-H_2_DCF-DA; Molecular Probes, Eugene, OR, USA). Differentiated podocytes on a glass dish were loaded with 5 µM CM-H_2_DCF-DA for 20 min at 37 °C. Excess dye was washed out using Krebs-Ringer bicarbonate buffer (KRB) solution. The fluorescence intensity was measured using the LSM 510 META laser-scanning confocal microscope.

### Fluorescent measurement of intracellular Ca^2+^ concentration

The intracellular Ca^2+^ concentration ([Ca^2+^]_*i*_) was measured as previously described. Briefly, [Ca^2+^]_*i*_ was measured using the Lambda DG-4 fluorescence measurement system (Sutter Instruments, Novato, CA, USA). Cells were placed on glass coverslips and loaded with Fura-2/AM in darkness for 30 min at 37 °C. After dye loading, the cells were washed and transferred to a perfusion chamber on a fluorescence microscope. Fura-2 signals were obtained by alternating excitation at 340 or 380 nm, and detecting emission at 510 nm. A normal physiological salt solution was used for bath perfusion that contained 135 mM NaCl, 5.4 mM KCl, 1 mM MgCl_2_, 2 mM CaCl_2_, 5 mM HEPES, and 10 mM glucose (pH 7.4). The data were analyzed using MetaFluor software (Sutter Instruments). All [Ca^2+^]_*i*_ measurements were performed at 37 °C using a heat controller (Warner Instruments, Hamden, CT, USA).

### Adhesion assay

Six-well plates were coated with type I collagen. Podocytes were harvested with trypsin/EDTA and resuspended in serum-free medium. Cells were allowed to attach at 37 °C for 1 h. Unbound cells were removed by washing twice with PBS. Attached cells were fixed with 4% PFA, and stained with crystal violet, and cell attachment was recorded for these experiments. Cell counts were obtained by averaging the cell number from five wells.

### Permeability assay

The permeability assay was assessed by measuring the passage of albumin across Collagen-coated Transwell^®^ (0.3 μm, Costar) seeded with podocytes transfected with MYH9 siRNA. Cells were then incubated in response to Ang II with or without losartan and NAC for 72 h. The upper compartment was refilled with 0.3 ml of DMEM and the lower compartment with 1 ml of 40 mg/ml BSA. The total protein concentration in the upper compartment was determined by the Bradford assay.

### Statistical analysis

Experimental values are presented as the mean ± SEM and SD. Statistical comparisons were made by Student’s *t*-test or one-way ANOVA, and *P* < 0.05 was considered significant.

## Supplementary information

supplementary information

unedited dataset 1
