# Supplementary material for: Angiotensin II-mediated MYH9 downregulation causes structural and functional podocyte injury in diabetic kidney disease
Source: Sci Rep. 2019 May 22;9:7679. doi: 10.1038/s41598-019-44194-3 (PMC6531474; doi:10.1038/s41598-019-44194-3)
Supplement: Supplementary file 1 — supplementary information [file 41598_2019_44194_MOESM1_ESM.docx]

**Angiotensin II-mediated MYH9 downregulation causes structural and functional podocyte injury in diabetic kidney disease**

Jeong Suk Kang^1,2^, Seung Joo Lee^1^, Ji-Hye Lee^3^, Ji-Hee Kim^4^, Seung Seob Son^1^, Seung-Kuy Cha^4^, Eun Soo Lee^5^, Choon Hee Chung^5^, and Eun Young Lee^1,2*^

^1^Department of Internal Medicine, Soonchunhyang University Cheonan Hospital, Cheonan, Korea

^2^Institute of Tissue Regeneration, College of Medicine, Soonchunhyang University, Cheonan, Korea

^3^Department of Pathology, Soonchunhyang University Cheonan Hospital, Cheonan, Korea; ^4^Department of Physiology, Yonsei University Wonju College of Medicine, Wonju, Korea; ^5^Department of Internal Medicine, Yonsei University Wonju College of Medicine, Wonju, Korea

^*^Correspondence and requests for materials should be addressed to E.Y.L. ([eylee@sch.ac.kr](mailto:eylee@sch.ac.kr))

**Supplementary Table 1: Clinical characteristics of patients with diabetic nephropathy.**


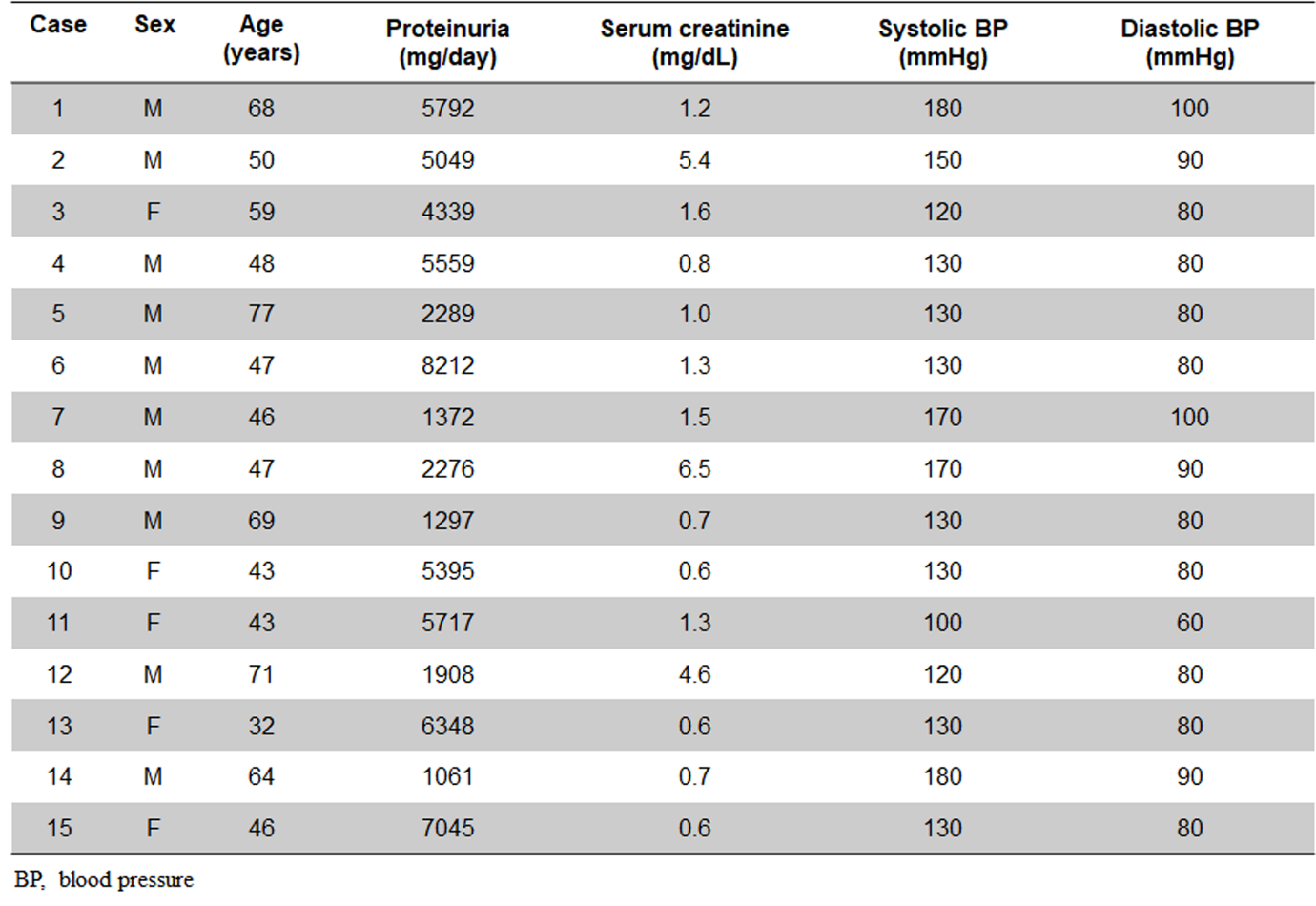


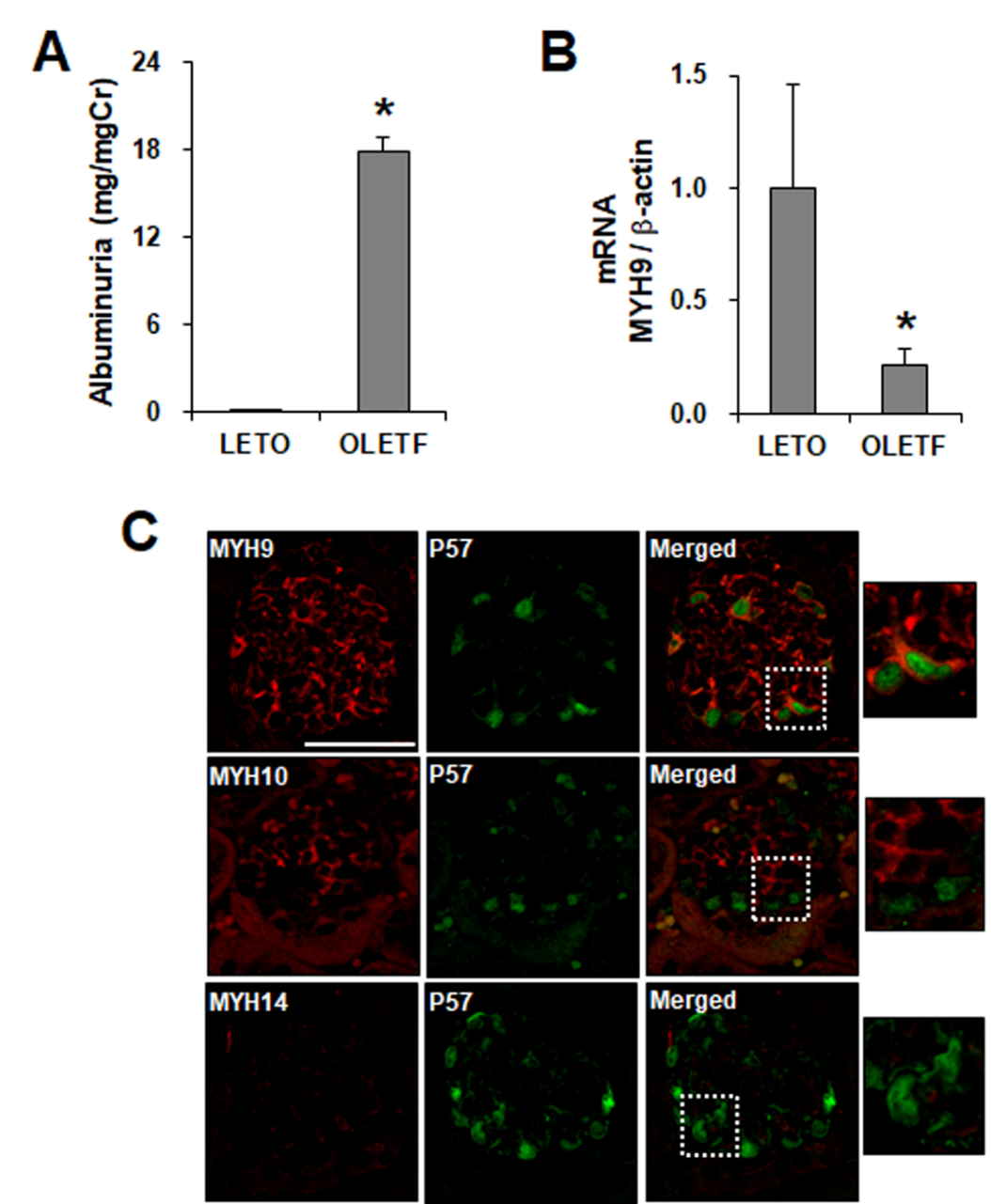
**Supplementary Figure S1: Downregulation of MYH9 in diabetic OLETF rat kidney and distribution of three NM II isoforms in glomeruli**. (A) OLETF rats displayed increased albuminuria (mg/mgCr) at 45 weeks of age (*n*=10 per group). (B) Decreased MYH9 mRNA expression was determined by real-time PCR, and corrected by β-actin mRNA levels in the same sample. (C) Paraffin-embedded mouse kidney sections were immunostained with antibodies, specific to MYH9, MYH10 and MYH14 to determine their localization in glomeruli (red). Podocyte-specific staining was performed with P57 (green). Unlike MYH9 in podocytes and mesangial cells, MYH10 was observed only in the mesangial cells. Dashed white boxes are enlarged images of representative podocytes. Magnification 40x; bar = 50. Data are presented as the means ± SEM. *P <0.05 versus nondiabetic LETO rats.


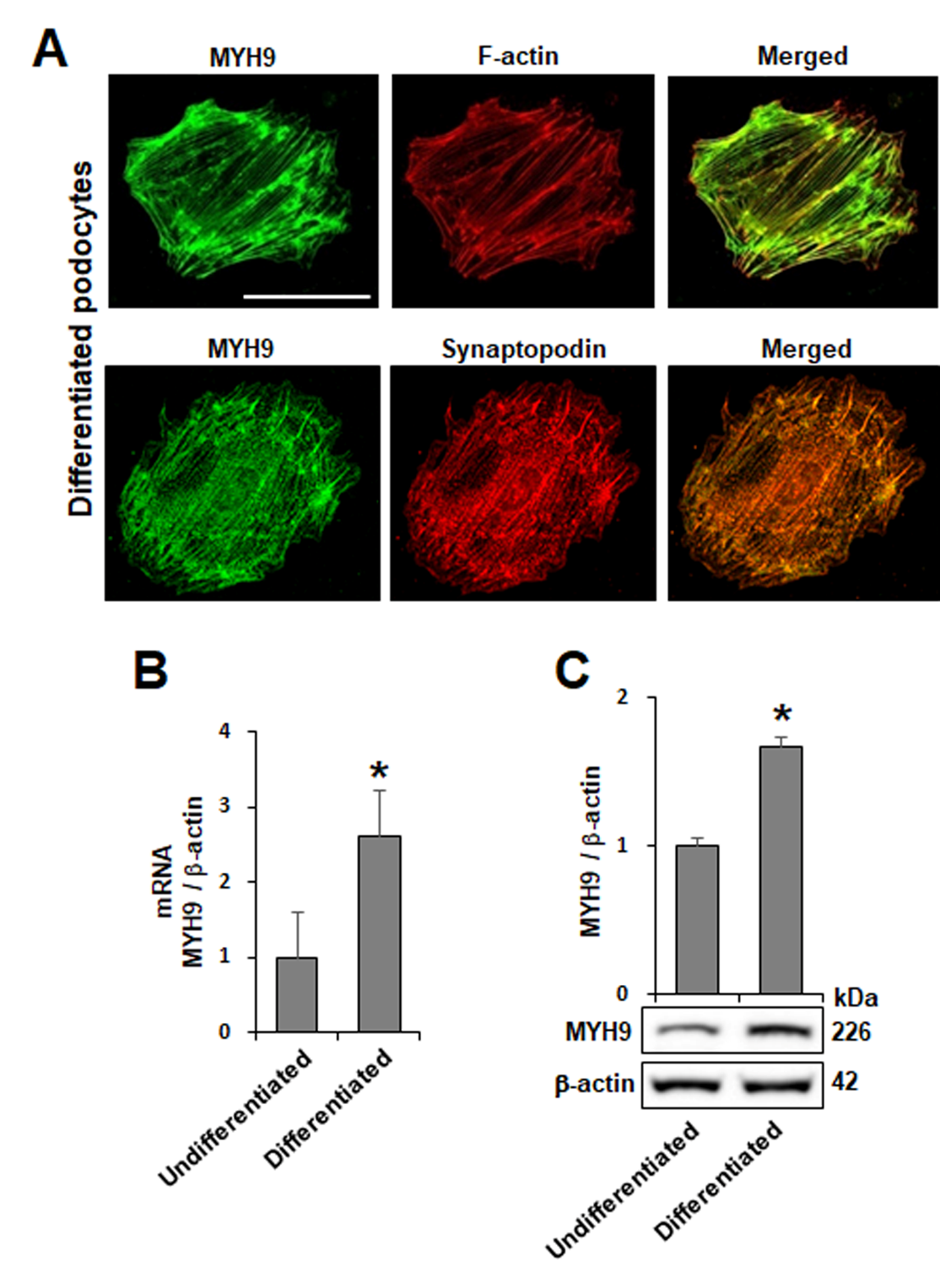
**Supplementary Figure S2: MYH9 localization along the actin stress fibers in differentiated podocytes.** (A) Differentiated podocytes were analyzed by immunofluorescence using anti-MYH9 (green), anti-rhodamine-phalloidin and anti-synaptopodin (red) antibodies, showing their colocalization along actin stress fibers. Magnification 40x; bar = 50. (B) Real-time PCR and (C) Western blot demonstrating increased MYH9 at the RNA and protein levels, respectively, in differentiated podocytes compared to undifferentiated cells. Data are presented as the means ± SEM. *P <0.05 versus undifferentiated podocytes.


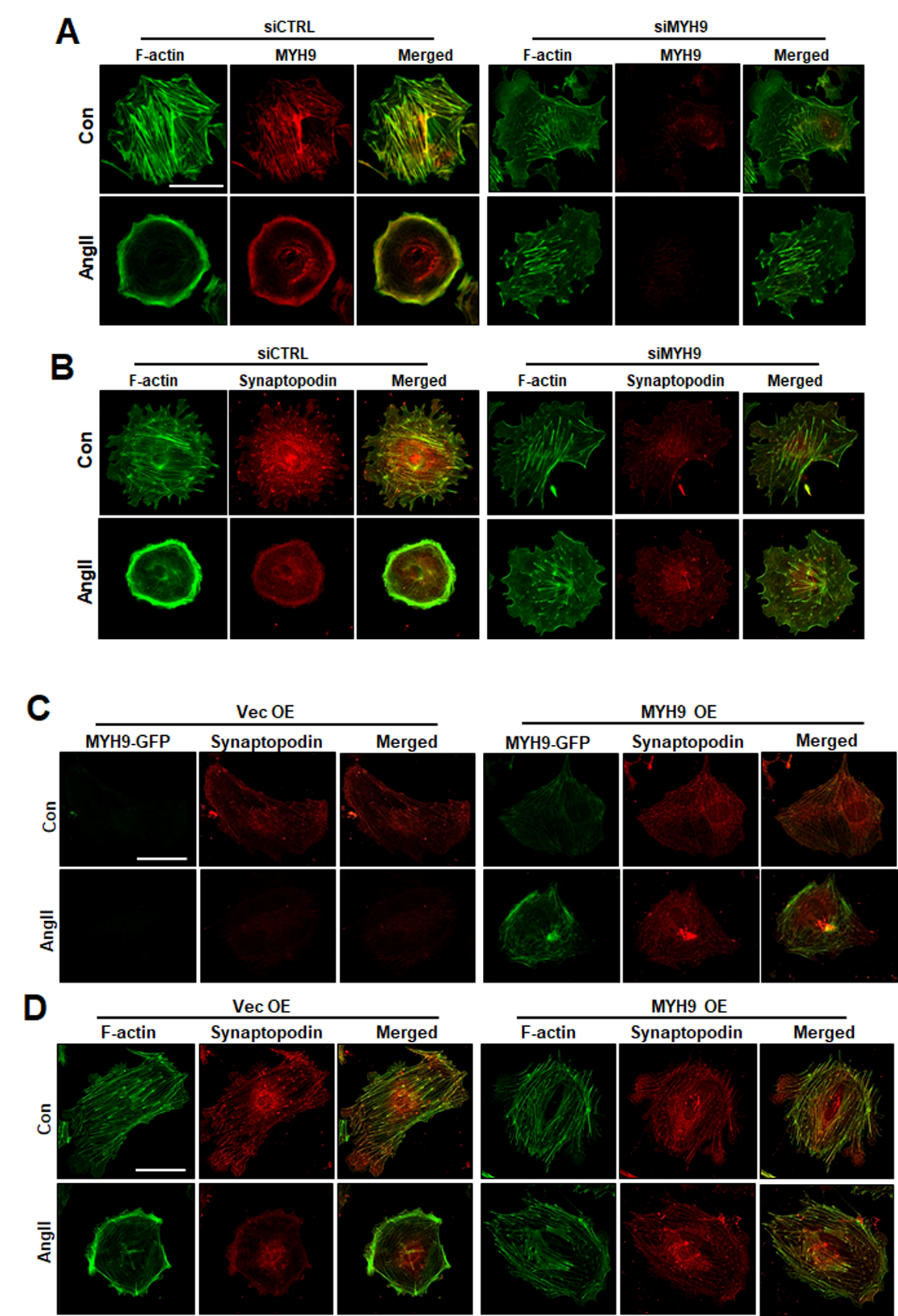


**Supplementary Figure S3: MYH9 and synaptopodin expression in MYH9-depleted or overexpressed podocytes.** (A) Cultured podocytes grown on coverslips were fixed with 4% PFA and immunolabeled with FITC-phalloidin and MYH9 (red) to confirm the reduction of MYH9 in MYH9 siRNA-transfected podocytes. Control cells display uniformed actin stress fibers. Treatment of podocytes with Ang II resulted in actin rearrangement and the loss of MYH9. MYH9 knockdown cells showed disorganized, shortened and decreased stress fibers and weak MYH9 staining. (B) Downregulation of synaptopodin in Ang II-treated or MYH9 knockout podocytes. (C, D) Podocytes transfected with GFP-MYH9 were analyzed by immunofluorescence with synaptopodin and FITC-phalloidin. MYH9 overexpressing podocytes (MYH9-GFP) showed nonreduced synaptopodin expression and uniformed actin stress fibers under Ang II treatment. Magnification 40x; bar = 50 μm. Similar results were obtained in three independent experiments.


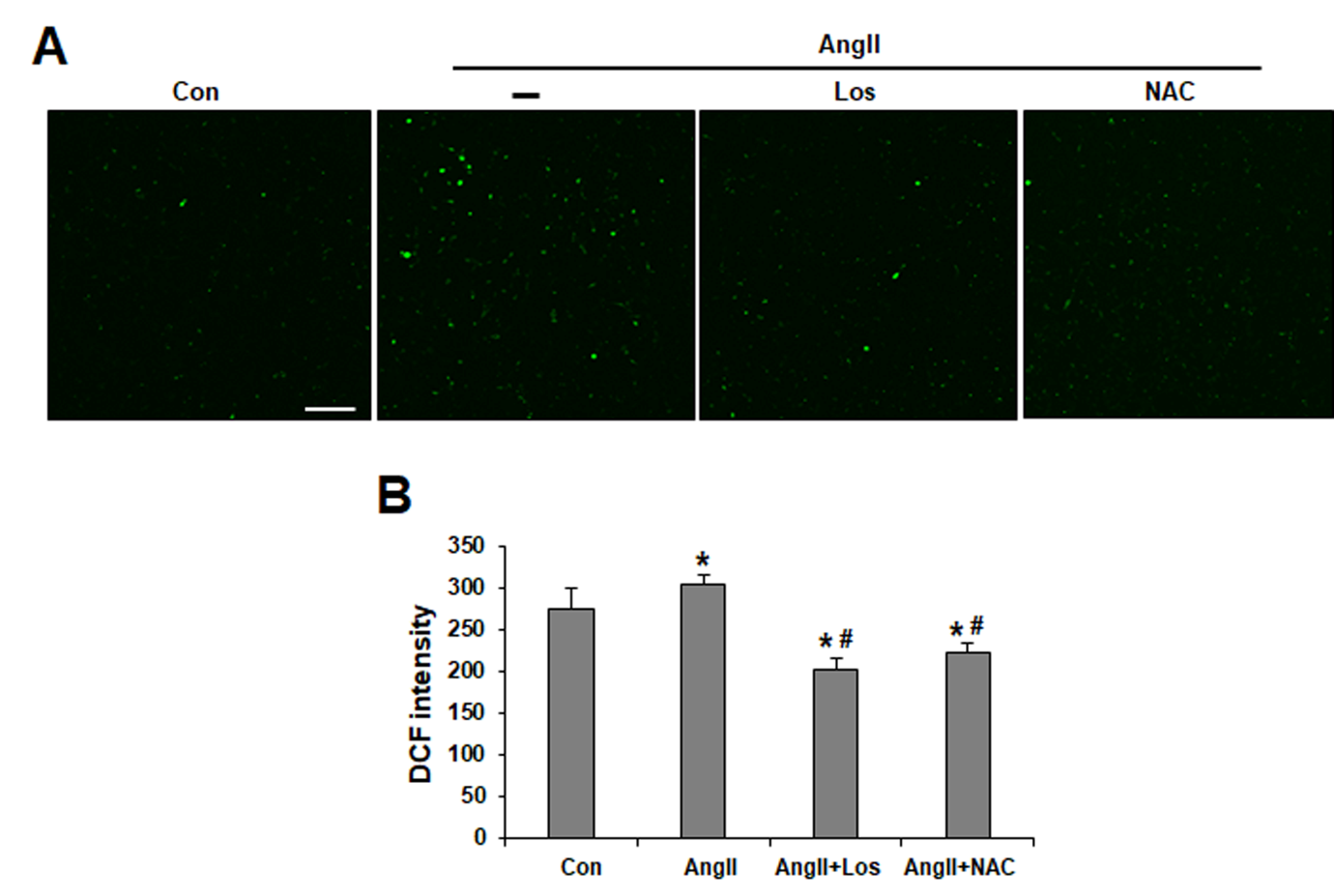


**Supplementary Figure S4: Effect of losartan and NAC on ROS generation.** (A) Representative images of ROS measurements in podocytes loaded with 2’7’-dichlorofluorescein (DCF) dye. Magnification 10x; bar = 300 μm. (B) Histogram analysis of the results shown in (A) for the comparison of average DCF intensities (*n*=4). ROS generation was measured using a fluorometer. Data are presented as the means ± SEM for three experiments. *P<0.05 compared with control. ^#^P<0.05 compared with Ang II-treated podocytes.
