# Supplementary material for: Angiotensin II-mediated MYH9 downregulation causes structural and functional podocyte injury in diabetic kidney disease
Source: Sci Rep. 2019 May 22;9:7679. doi: 10.1038/s41598-019-44194-3 (PMC6531474; doi:10.1038/s41598-019-44194-3)

Figure 1A

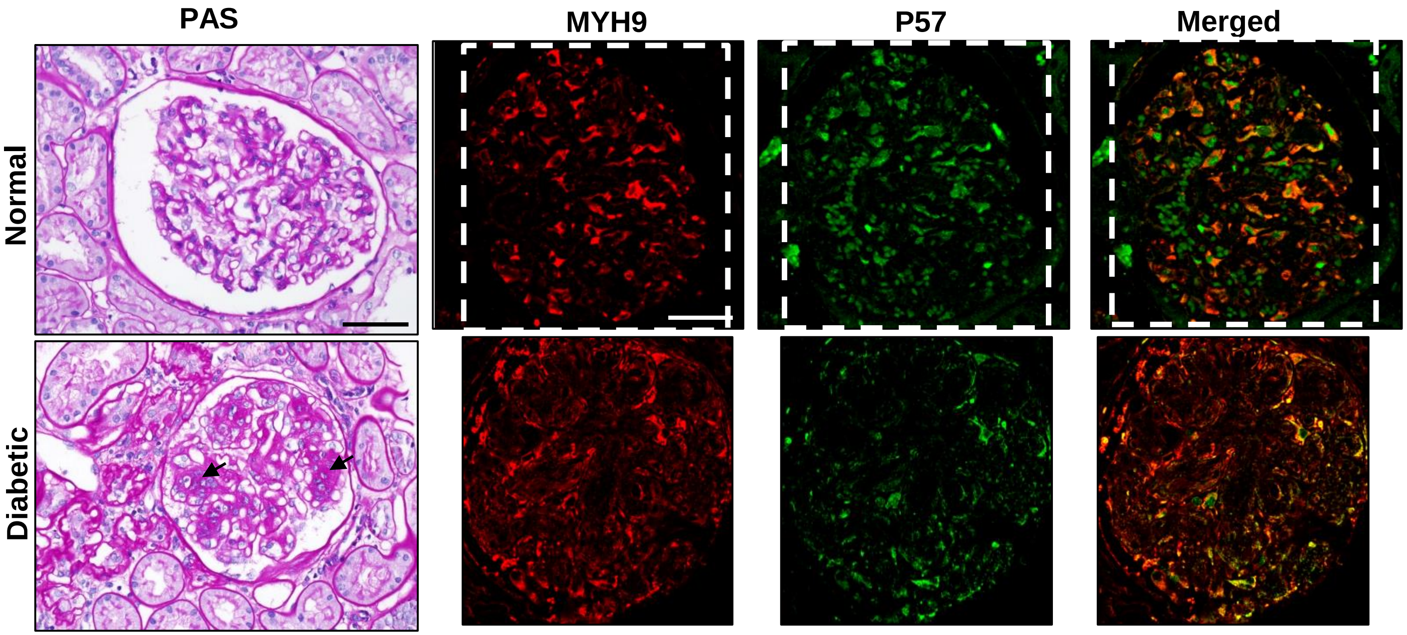

Figure 1D

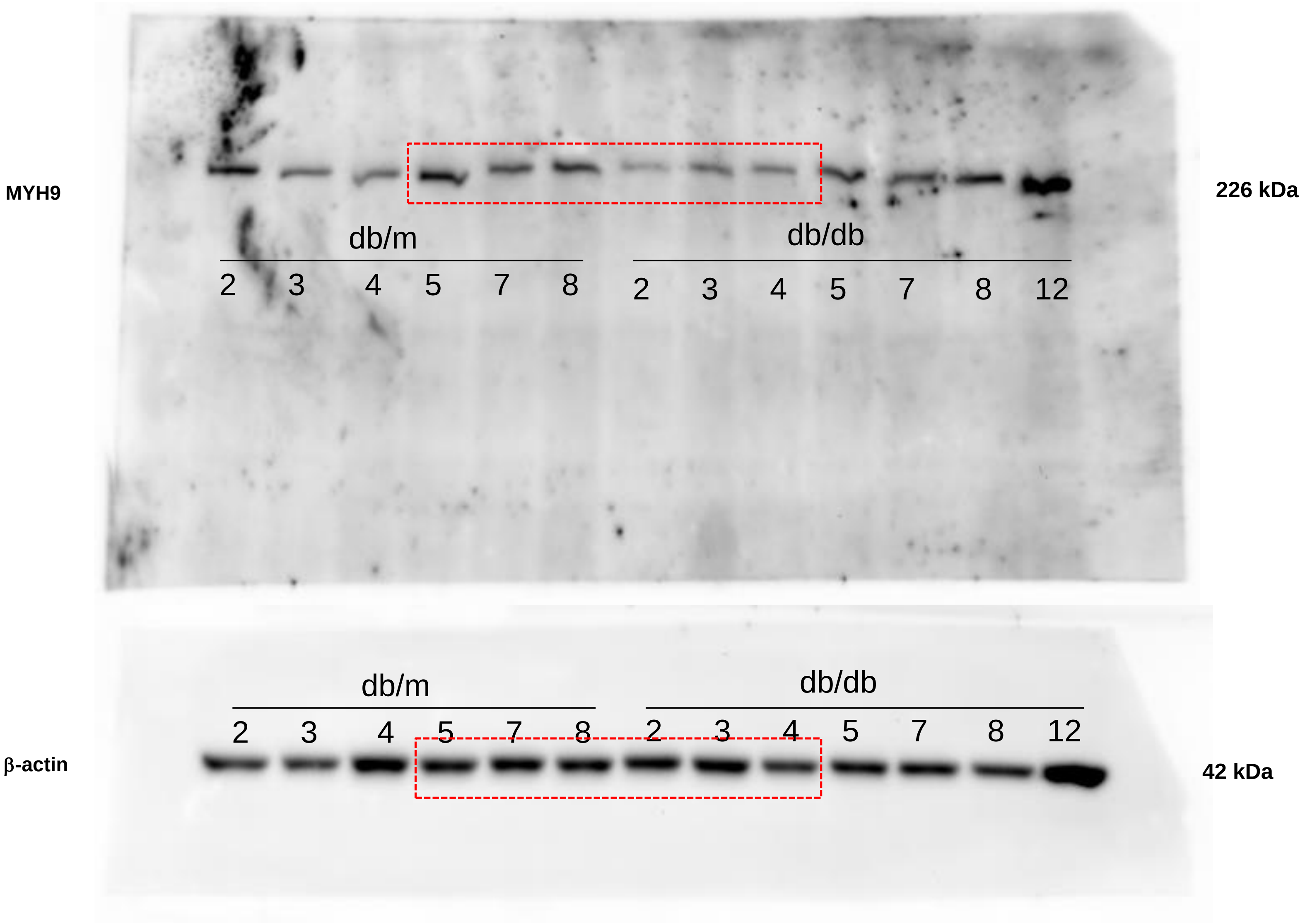

MYH9
db/m
2 3 4 5 7 8
db/db
2 3 4 5 7 8 12
226 kDa
β-actin
db/m
2 3 4 5 7 8
db/db
2 3 4 5 7 8 12
42 kDa

Figure 1E

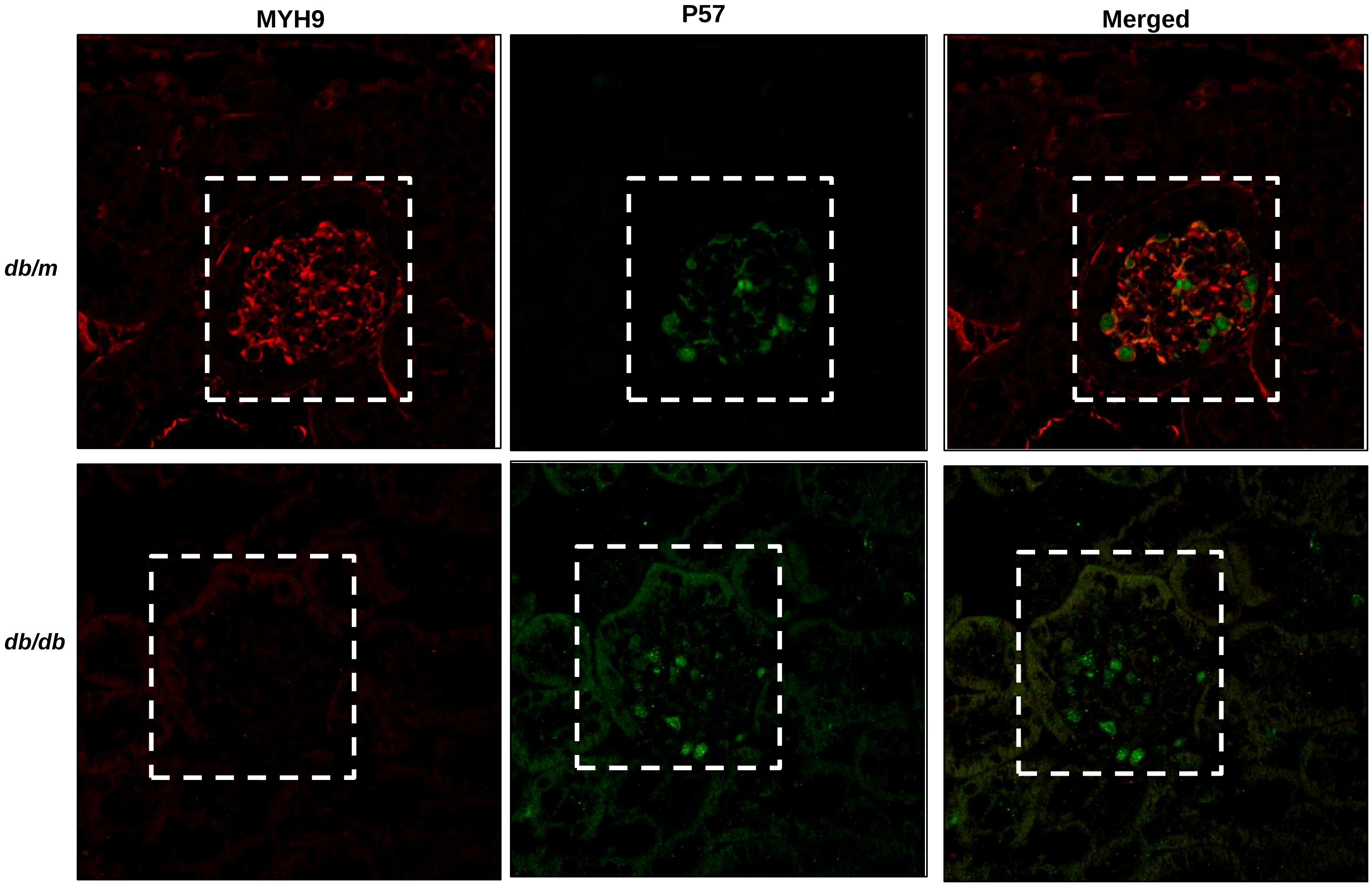

Figure2A

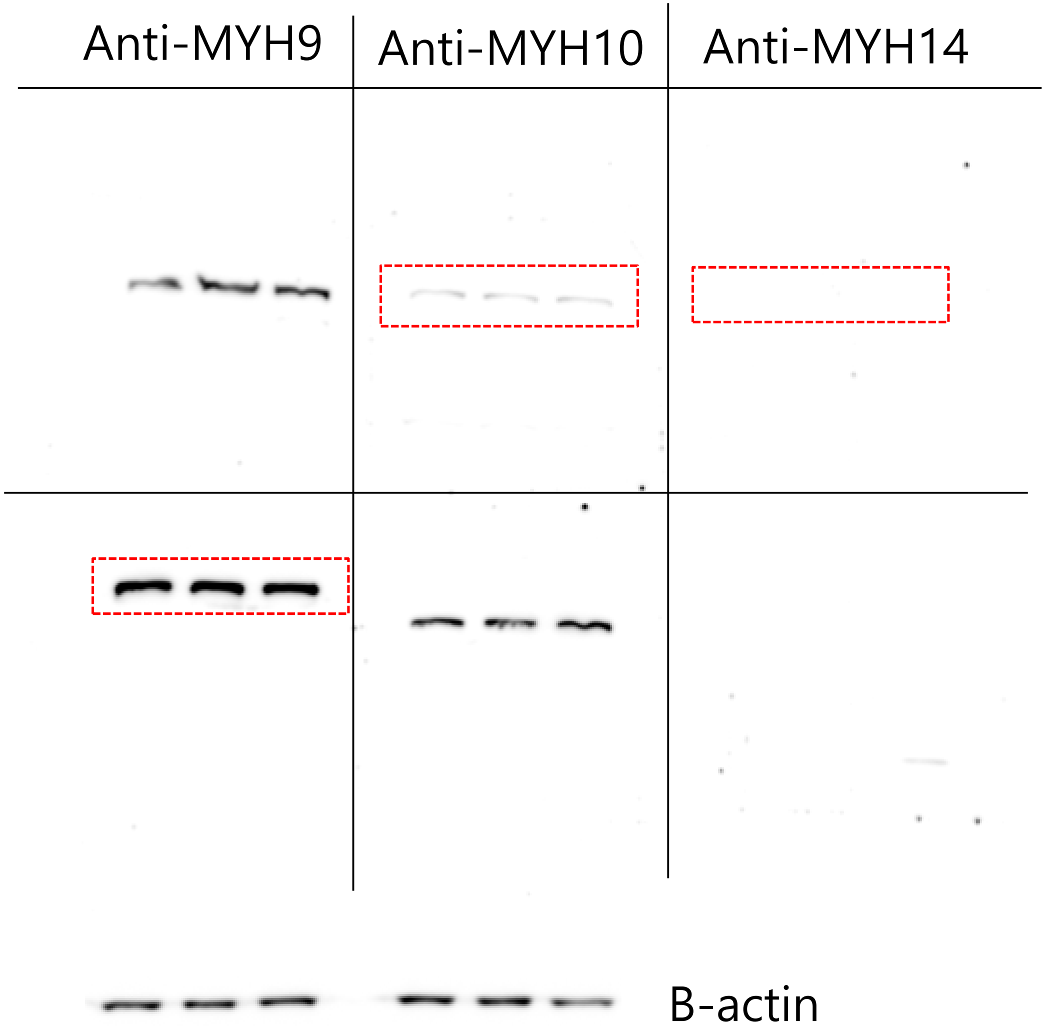

Figure2B

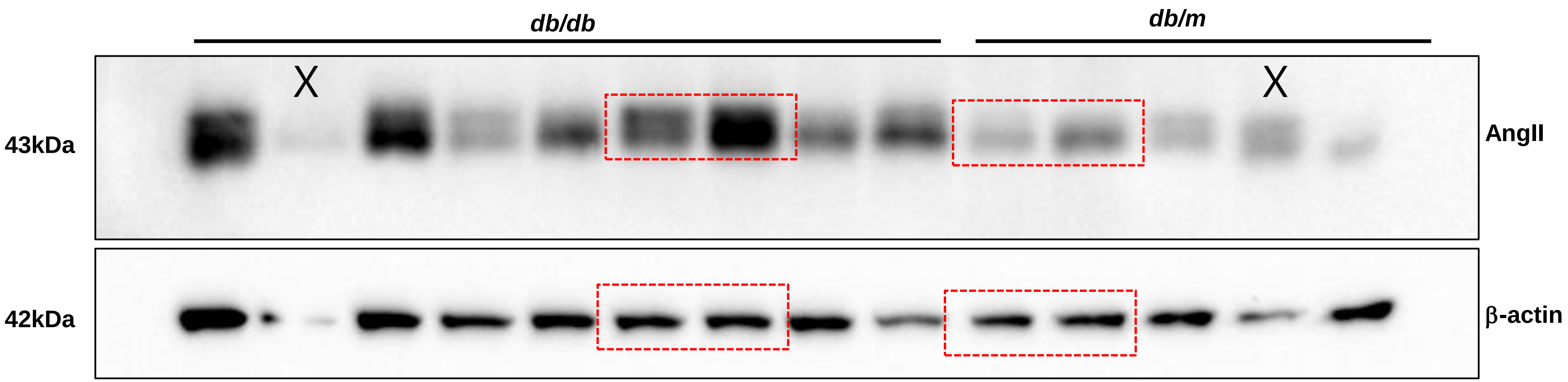

Figure 2E

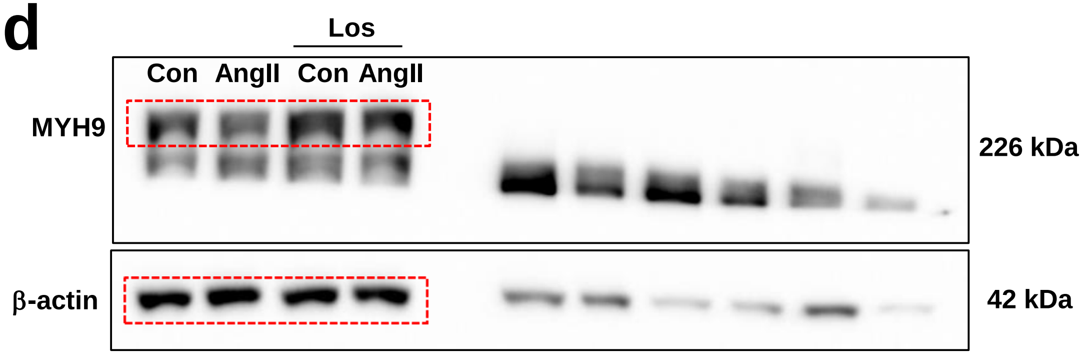

Figure 3B

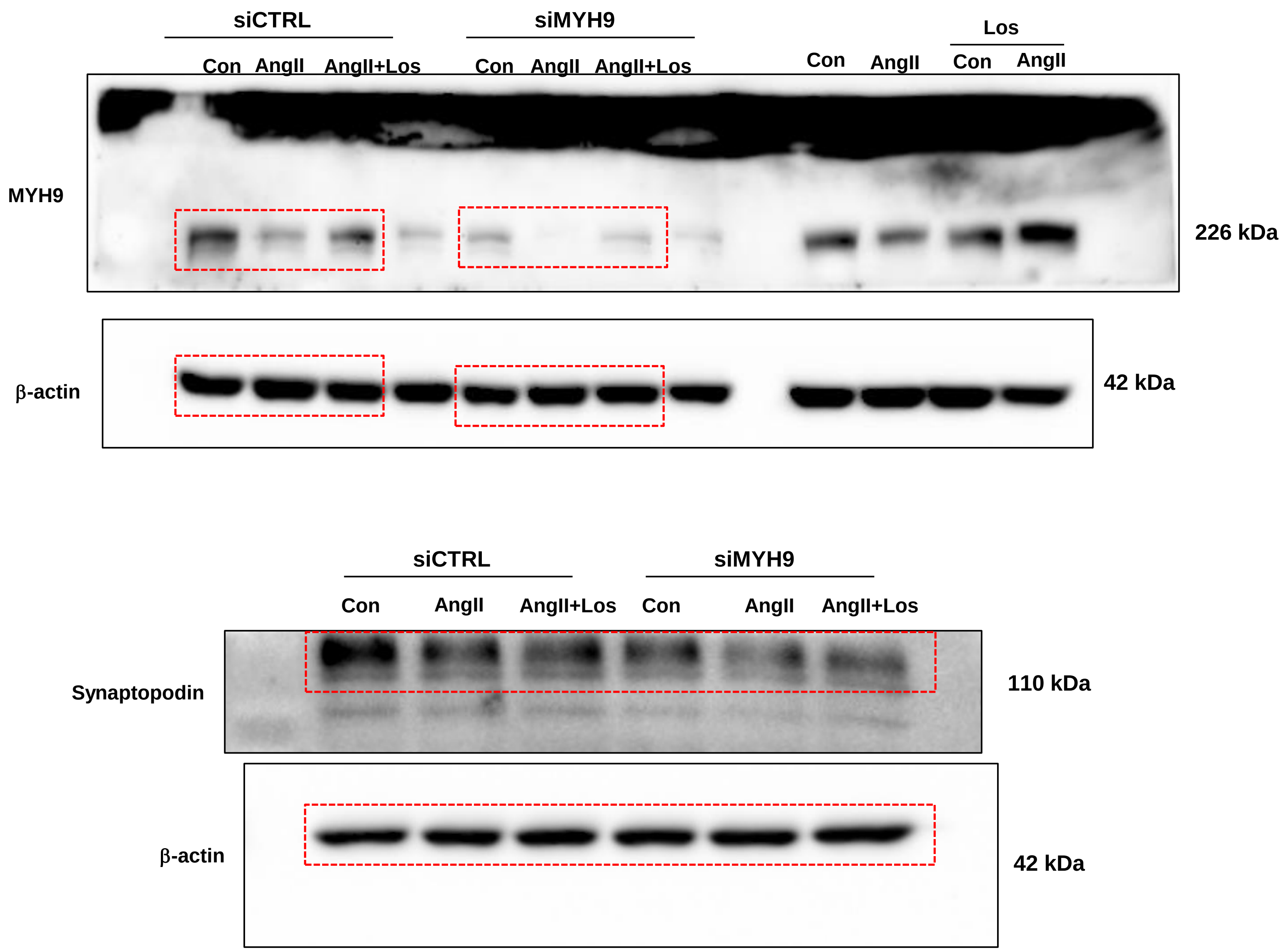
siCTRL
siMYH9
Los
Con
AngII
AngII+Los
Con
AngII
AngII+Los
Con
AngII
Con
AngII
MYH9
226 kDa
β-actin
42 kDa
siCTRL
siMYH9
Con
AngII
AngII+Los
Con
AngII
AngII+Los
Synaptopodin
110 kDa
β-actin
42 kDa

Figure 3C

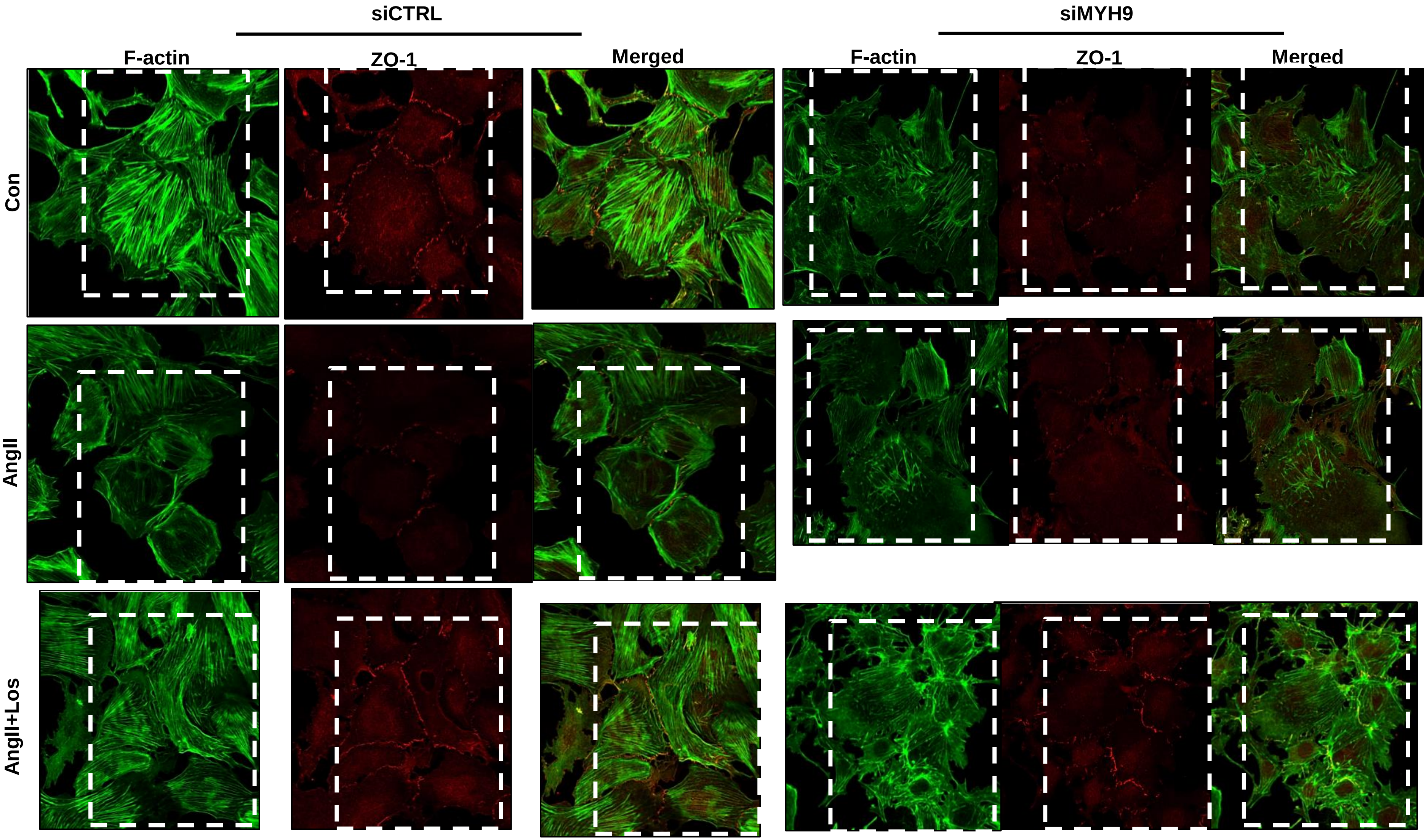
siCTRL
siMYH9
F-actin
ZO-1
Merged
F-actin
ZO-1
Merged
Con
AngII
AngII+Los

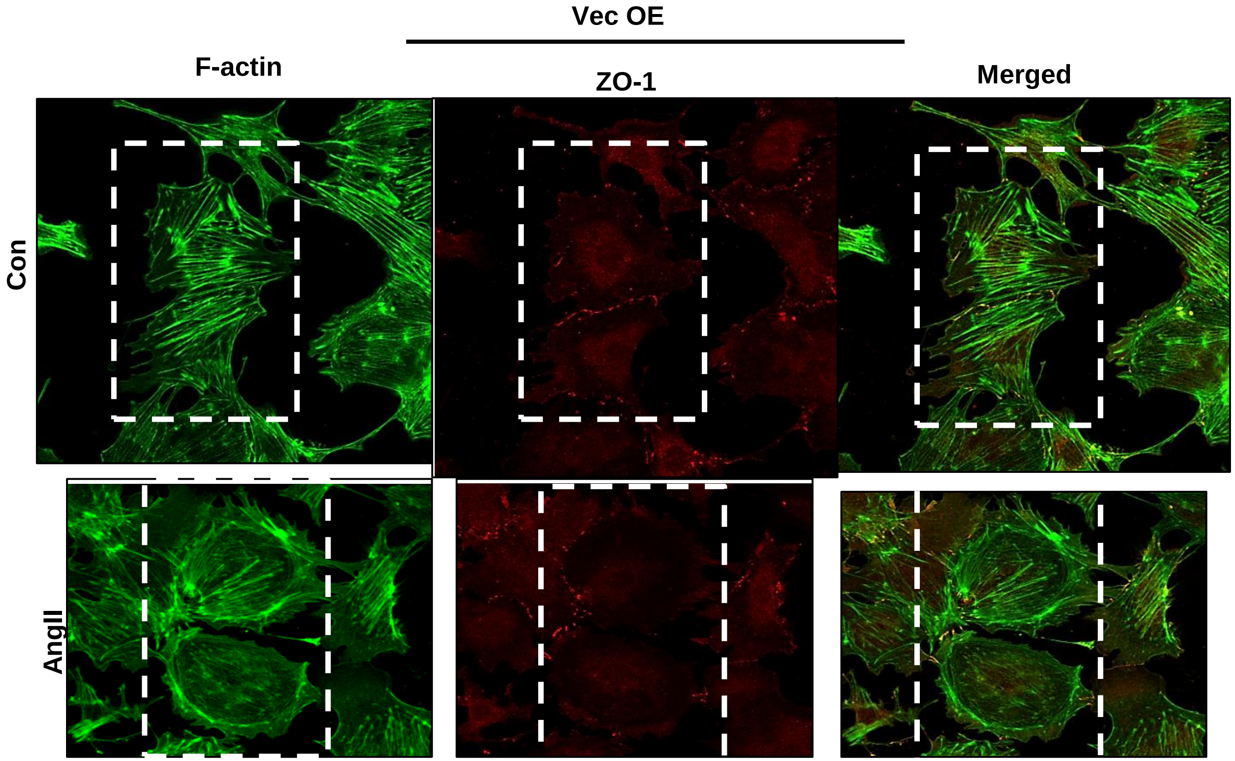

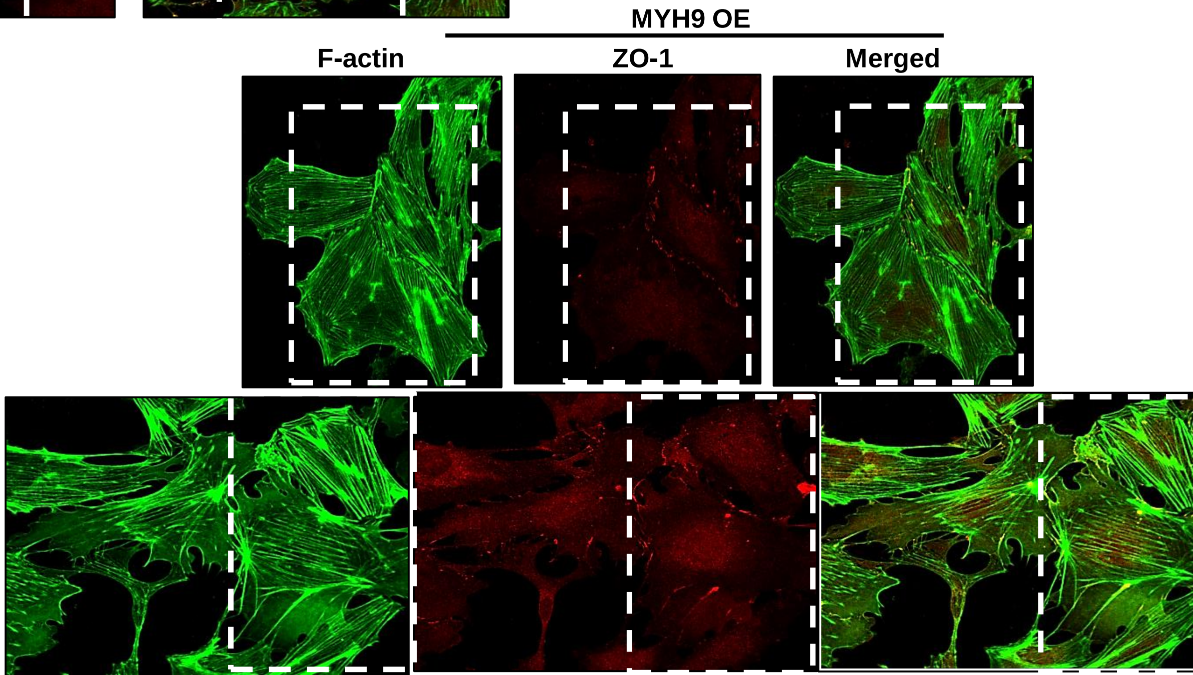

Figure 3D

Figure 4C

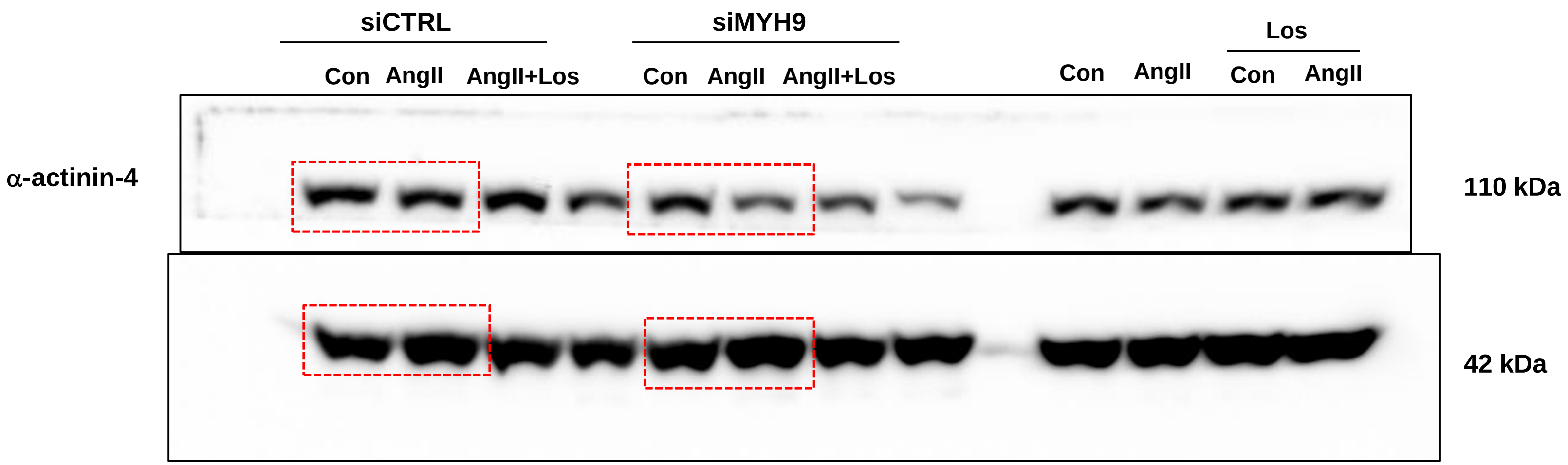
siCTRL
siMYH9
Los
Con
AngII
AngII+Los
Con
AngII
AngII+Los
Con
AngII
Con
AngII
α-actinin-4
110 kDa
42 kDa

Figure 4C

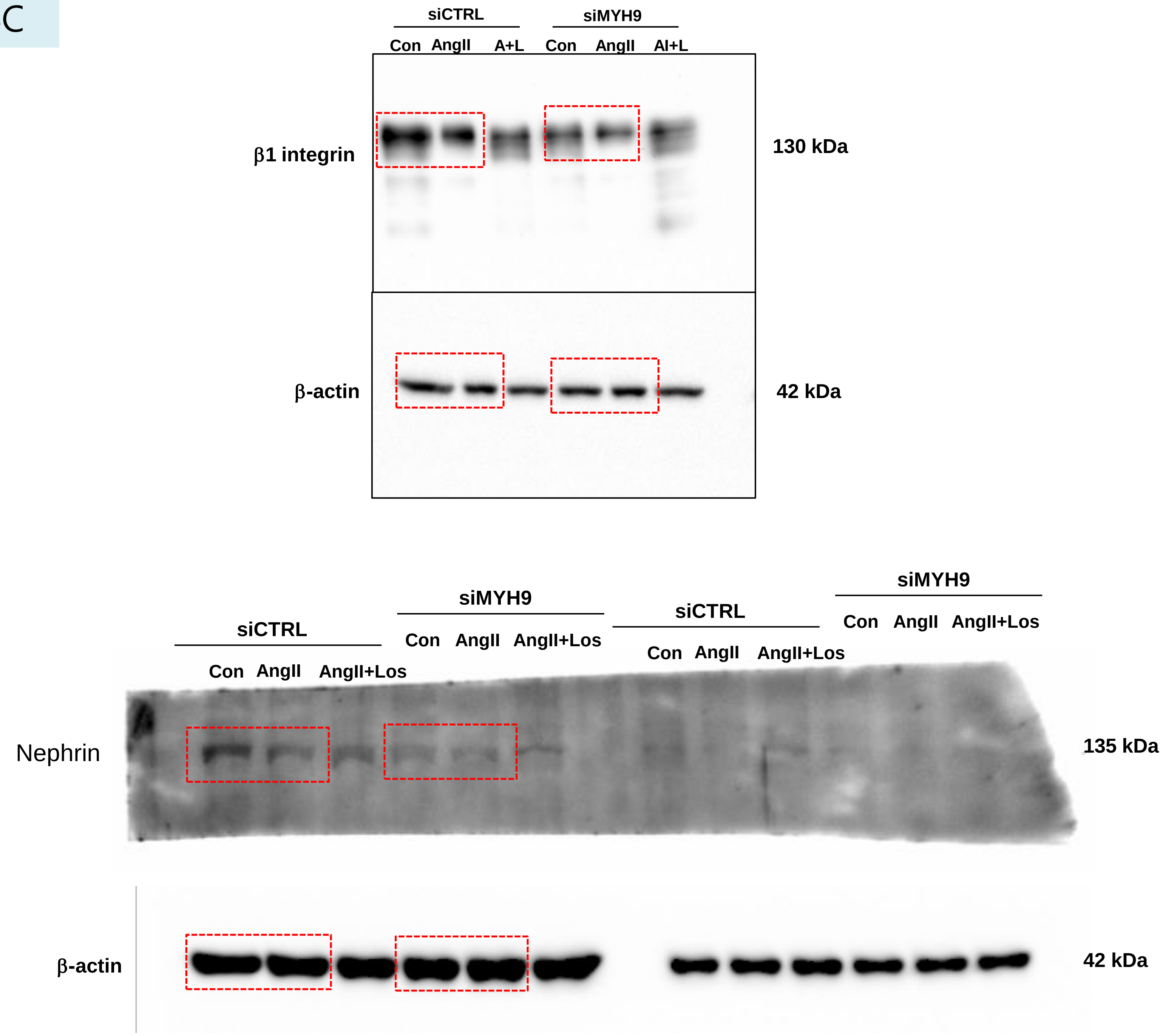

siCTRL
siMYH9
Con
AngII
A+L
Con
AngII
AI+L
β1 integrin
130 kDa
β-actin
42 kDa
siCTRL
siMYH9
siCTRL
siMYH9
Con
AngII
AngII+Los
Nephrin
135 kDa
β-actin
42 kDa

Figure 5A

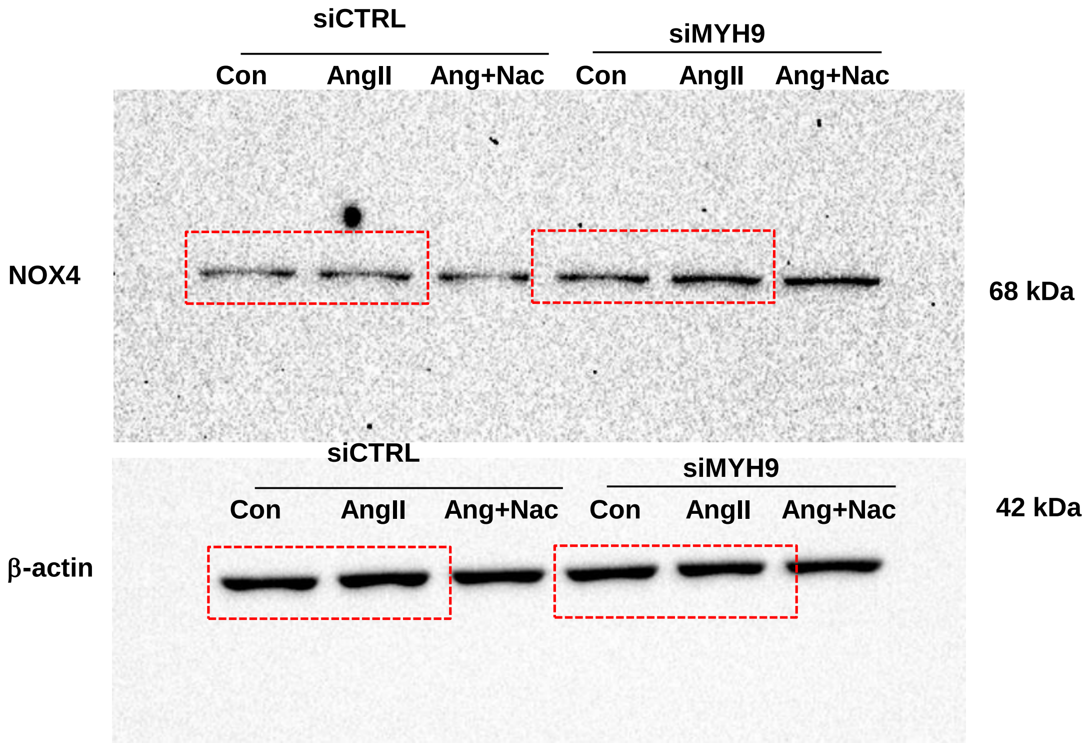

Figure 5C

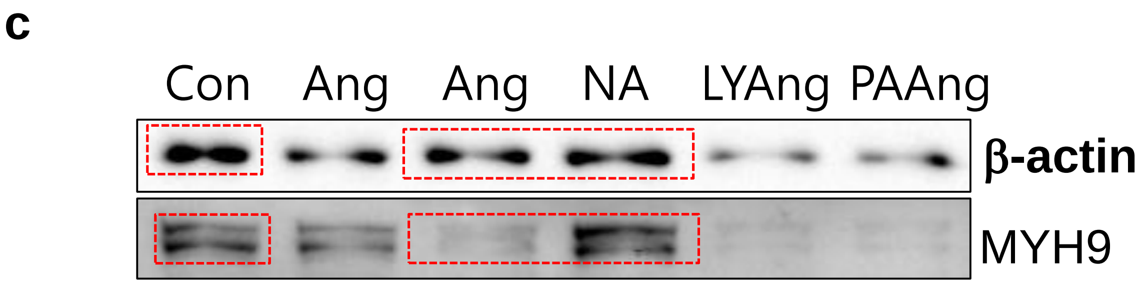

Figure 5D

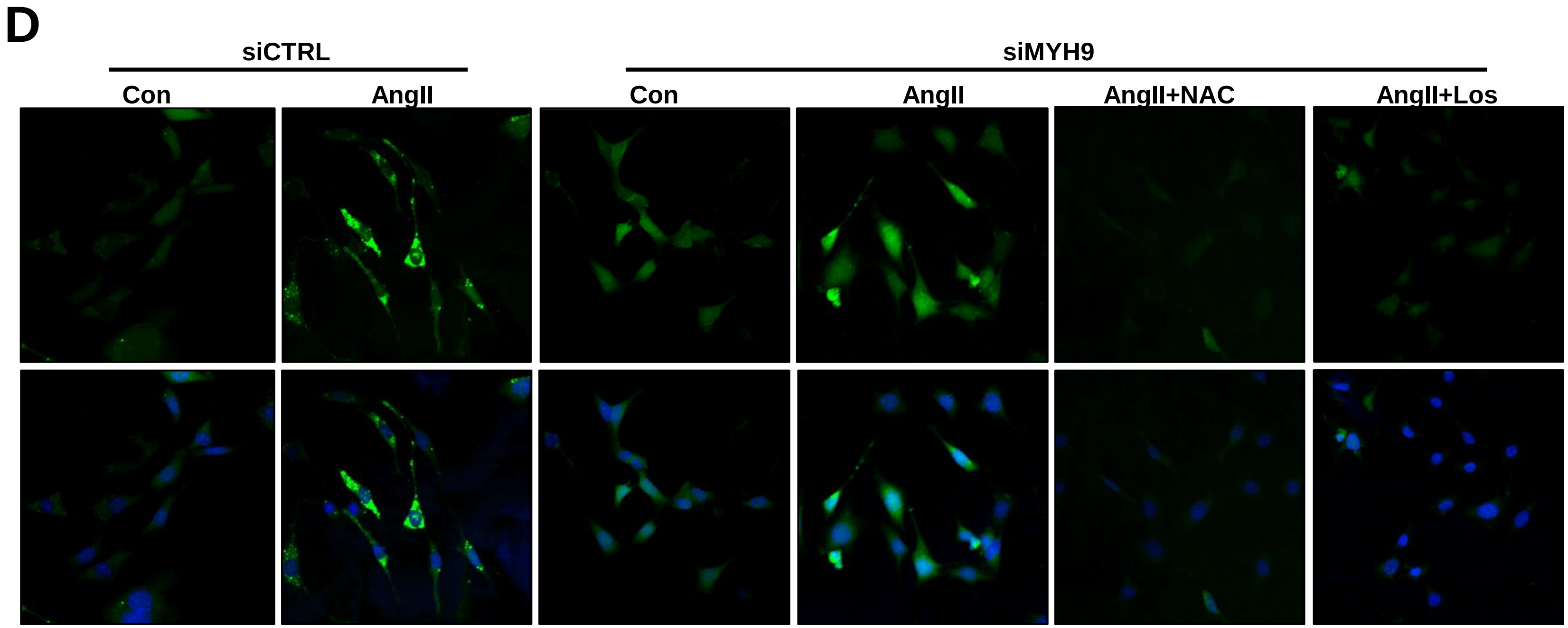

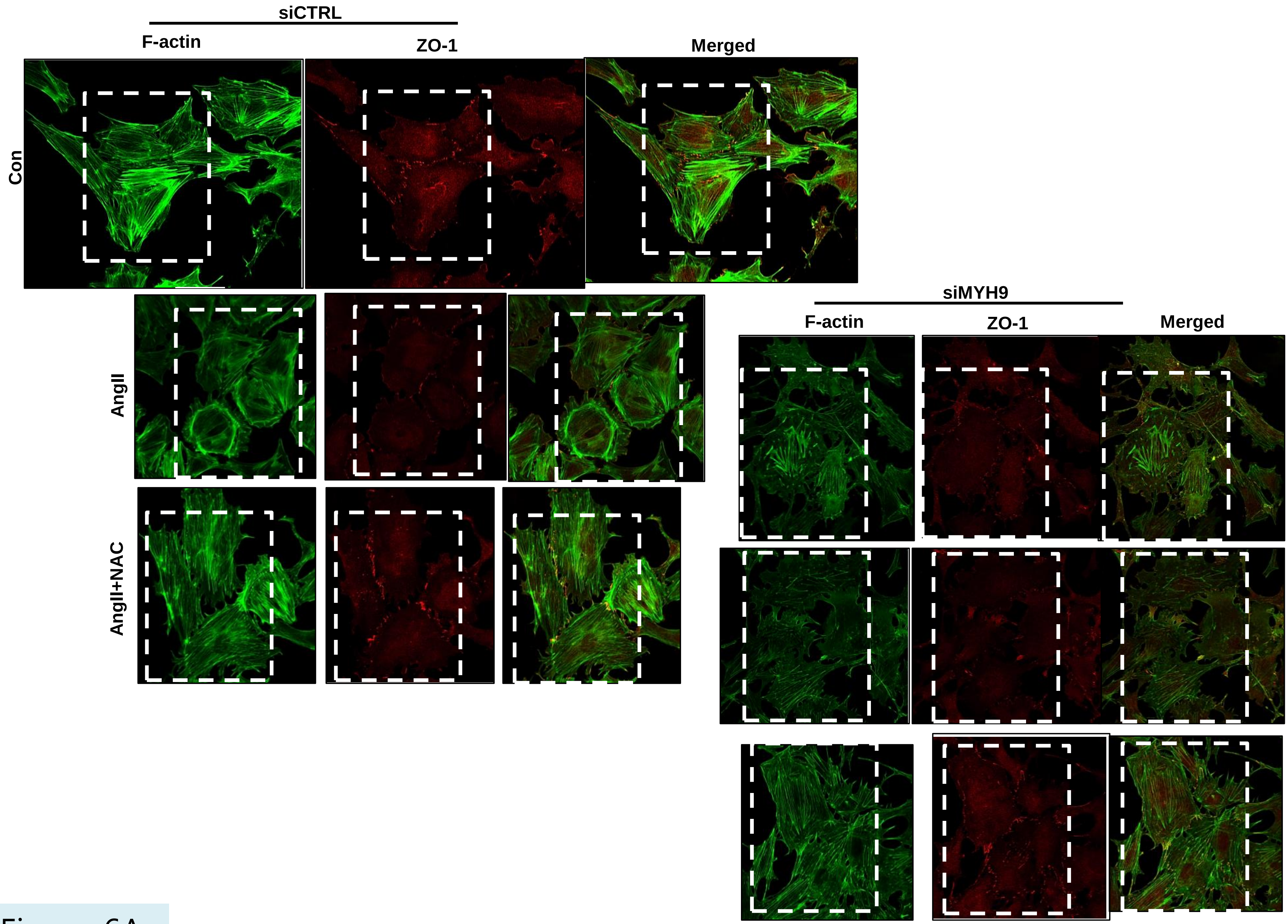
siCTRL
F-actin
ZO-1
Merged
Con
AngII
AngII+NAC
siMYH9
F-actin
ZO-1
Merged

Figure 6A

Figure 8F

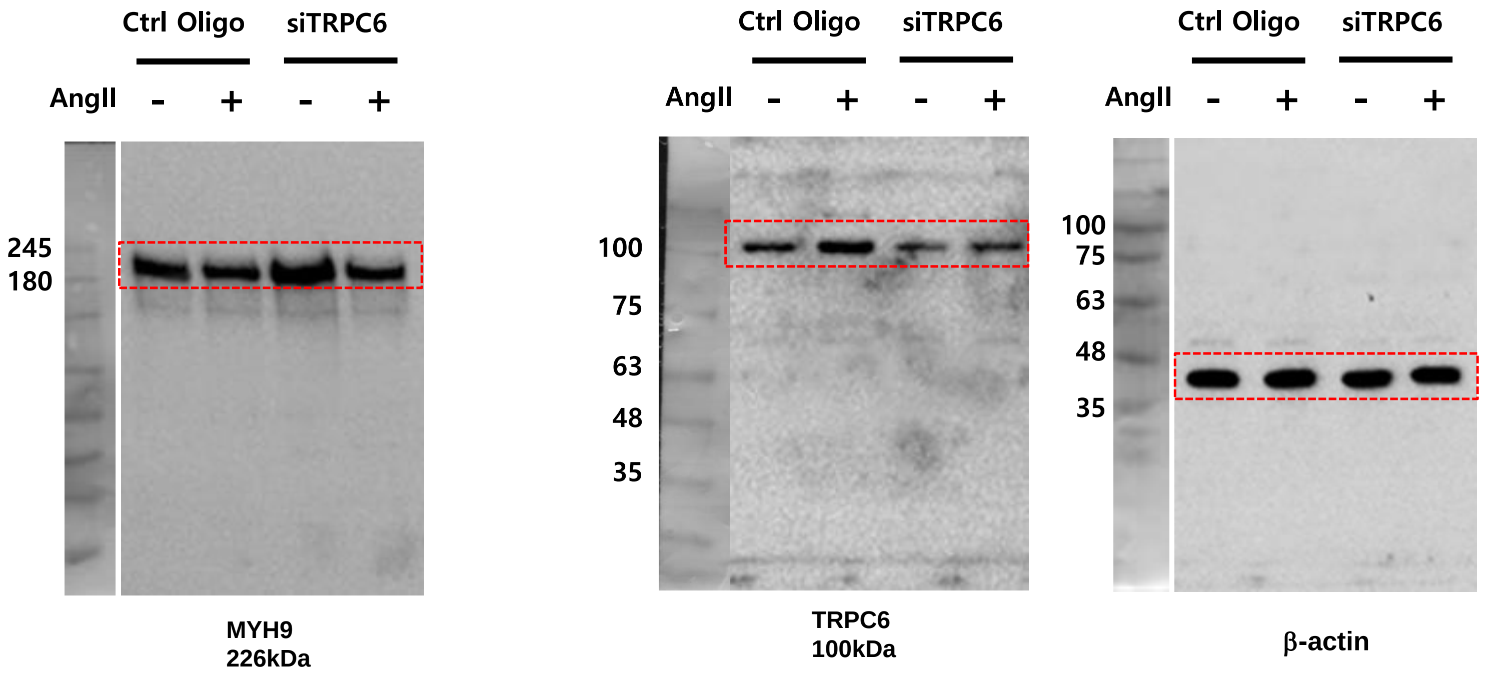

Ctrl Oligo
siTRPC6
AngII - + - +
245
180
MYH9
226kDa
Ctrl Oligo
siTRPC6
AngII - + - +
100
75
63
48
35
TRPC6
100kDa
Ctrl Oligo
siTRPC6
AngII - + - +
100
75
63
48
35
β-actin

SFigure 1C

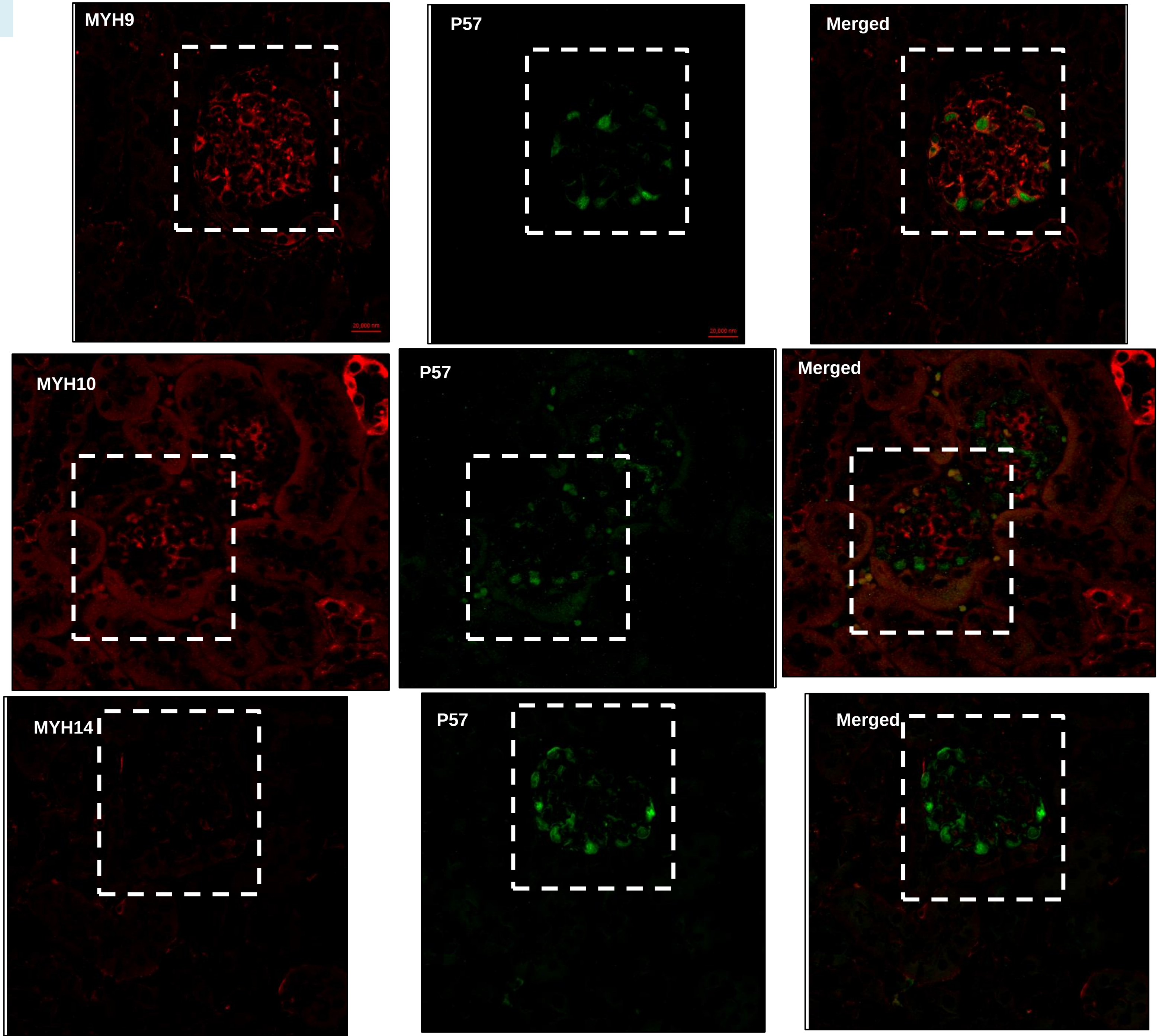

SFigure 2A

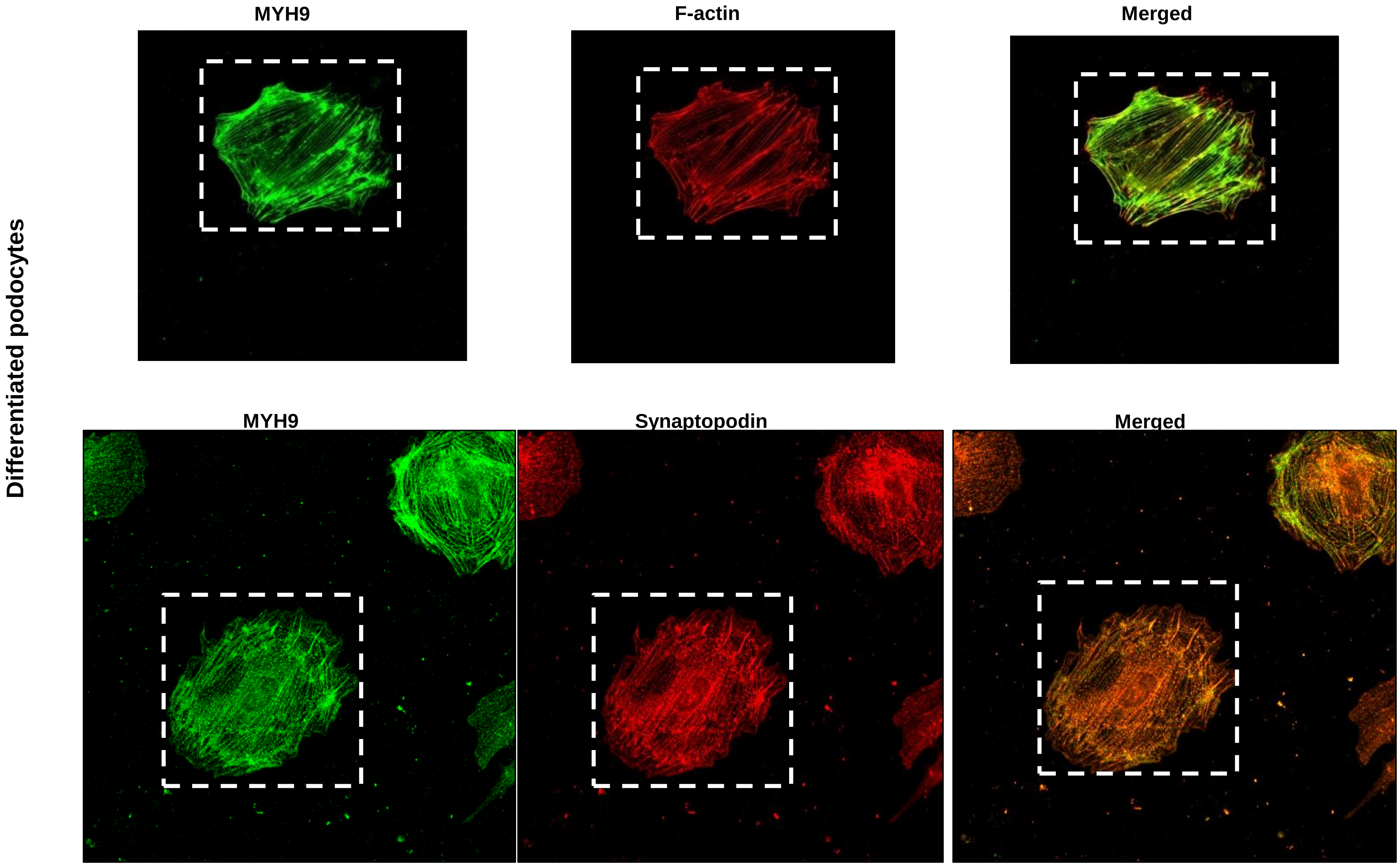
MYH9
F-actin
Merged
Differentiated podocytes
MYH9
Synaptopodin
Merged

SFigure 3A

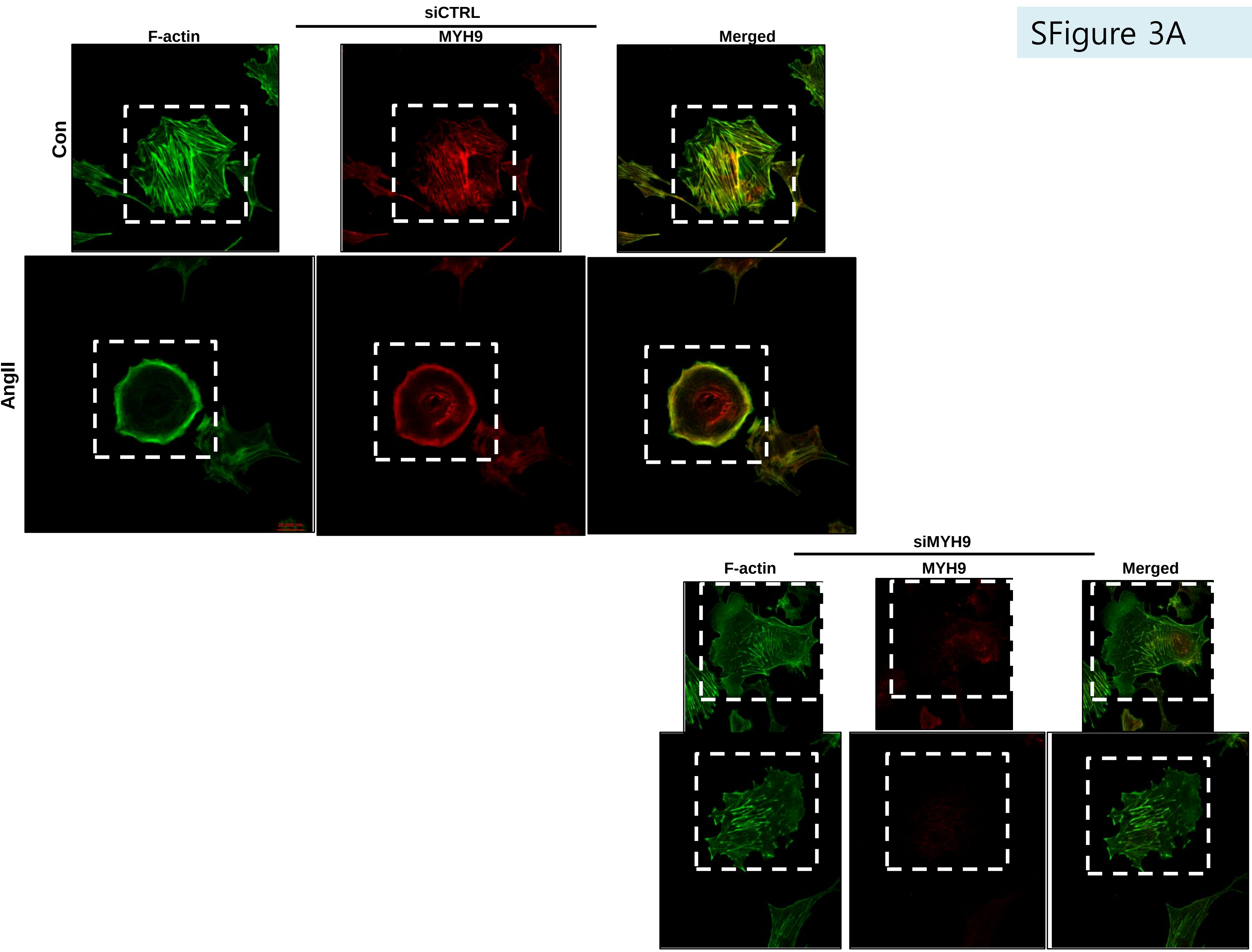
siCTRL
F-actin
MYH9
Merged
Con
AngII
siMYH9
F-actin
MYH9
Merged

SFigure 3B

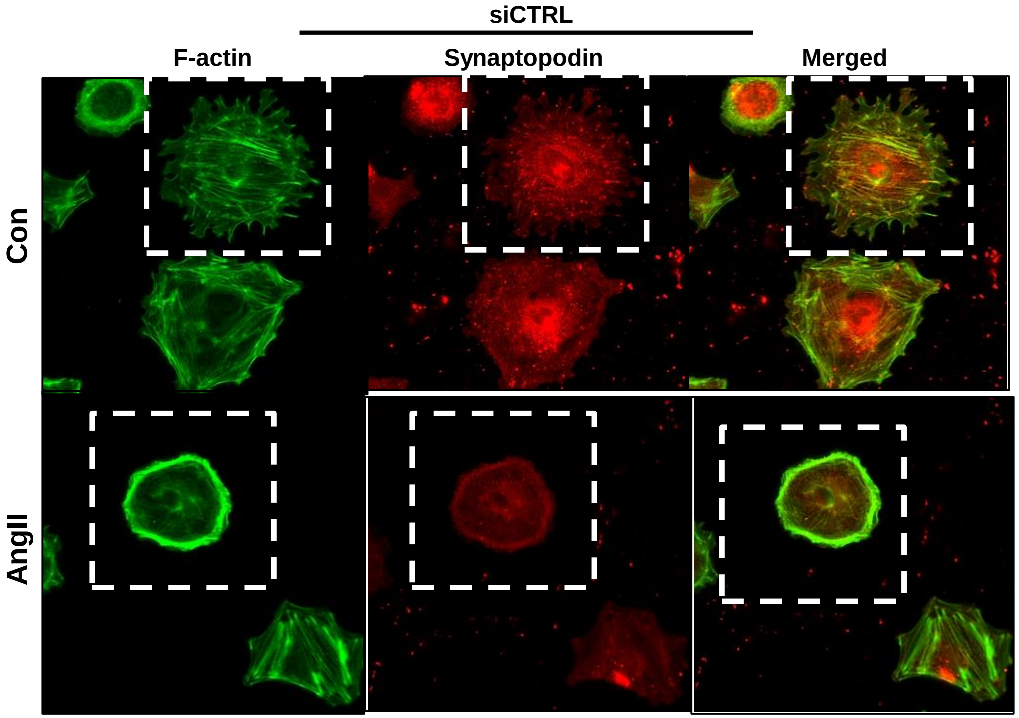
siCTRL
F-actin
Synaptopodin
Merged
Con
AngII

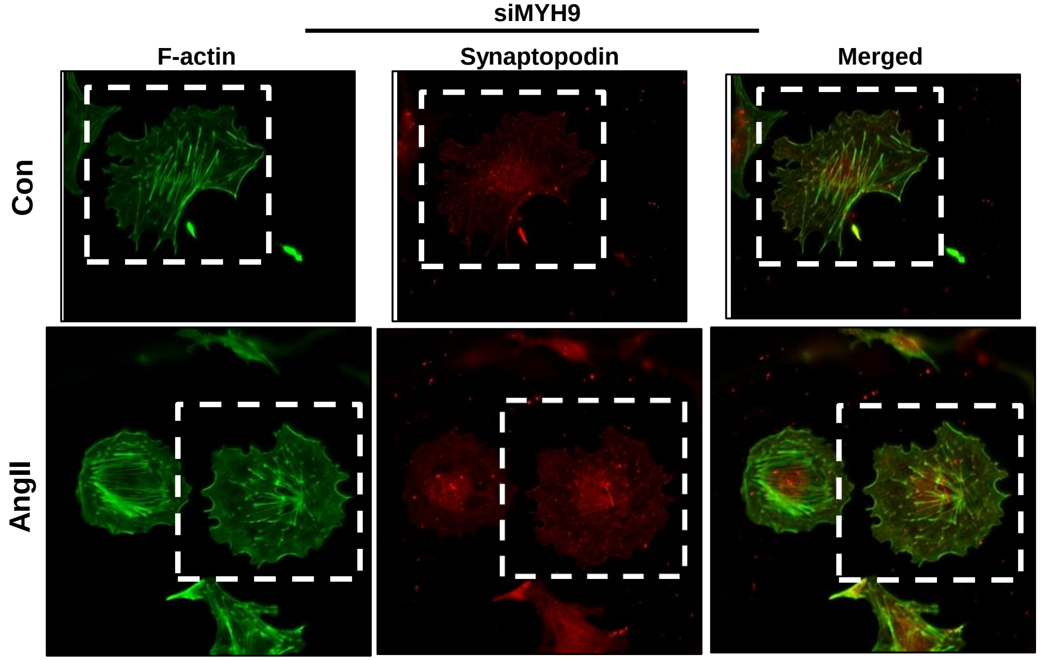
siMYH9
F-actin
Synaptopodin
Merged
Con
AngII

SFigure 3C

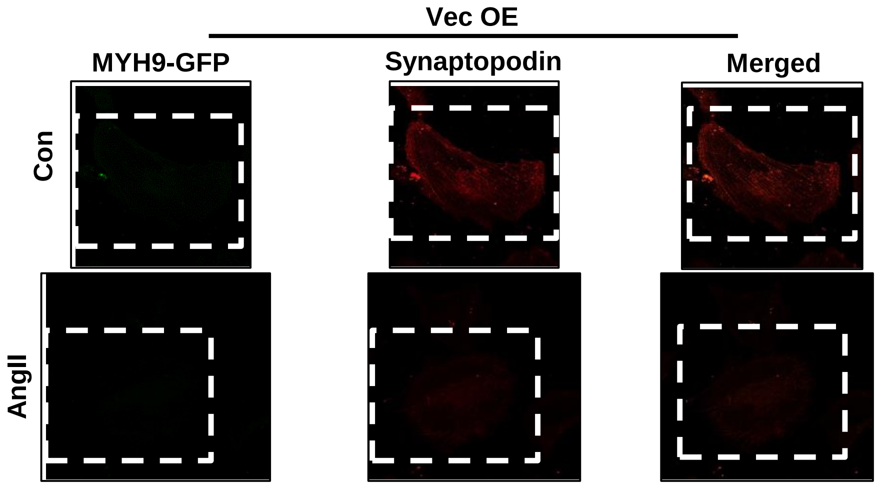
Vec OE
MYH9-GFP
Synaptopodin
Merged
Con
AngII

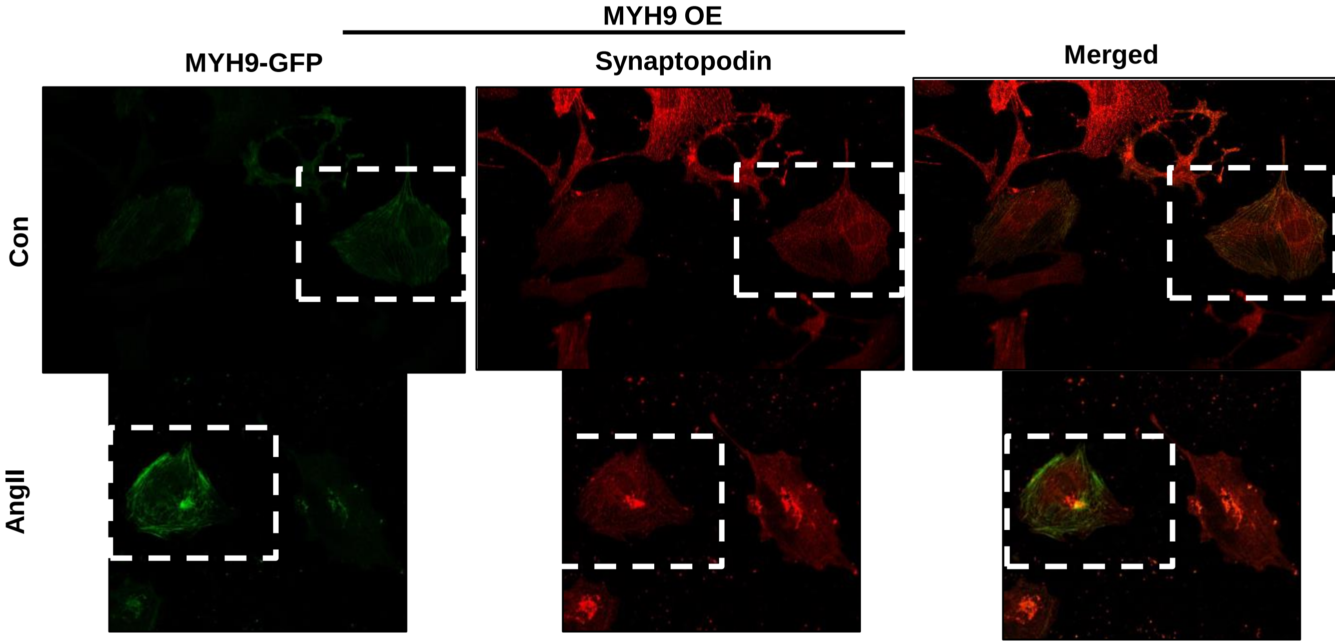
MYH9 OE
MYH9-GFP
Synaptopodin
Merged
Con
AngII

SFigure 3D

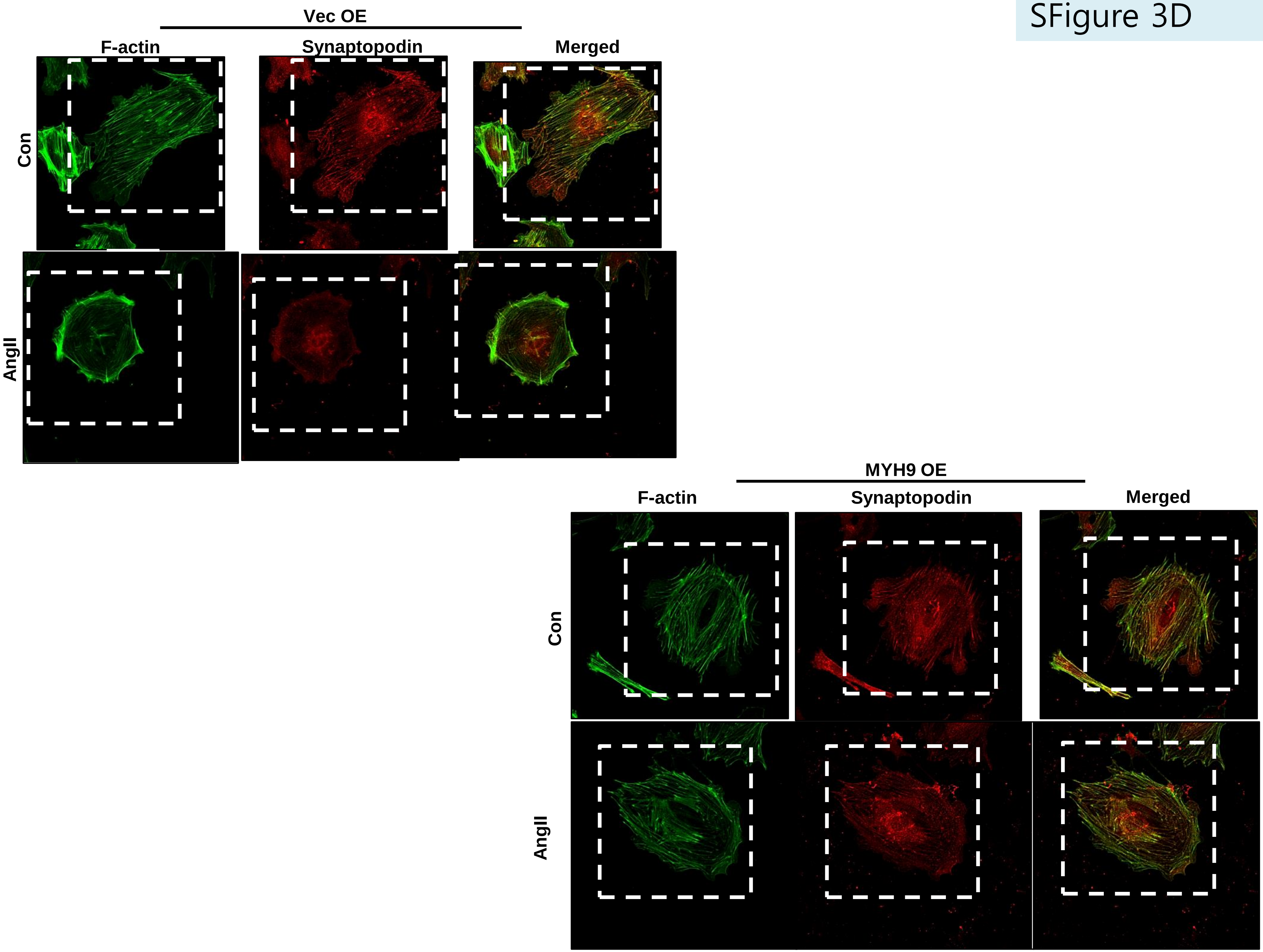
Vec OE
F-actin
Synaptopodin
Merged
Con
AngII
MYH9 OE
F-actin
Synaptopodin
Merged
Con
AngII

SFigure 4A

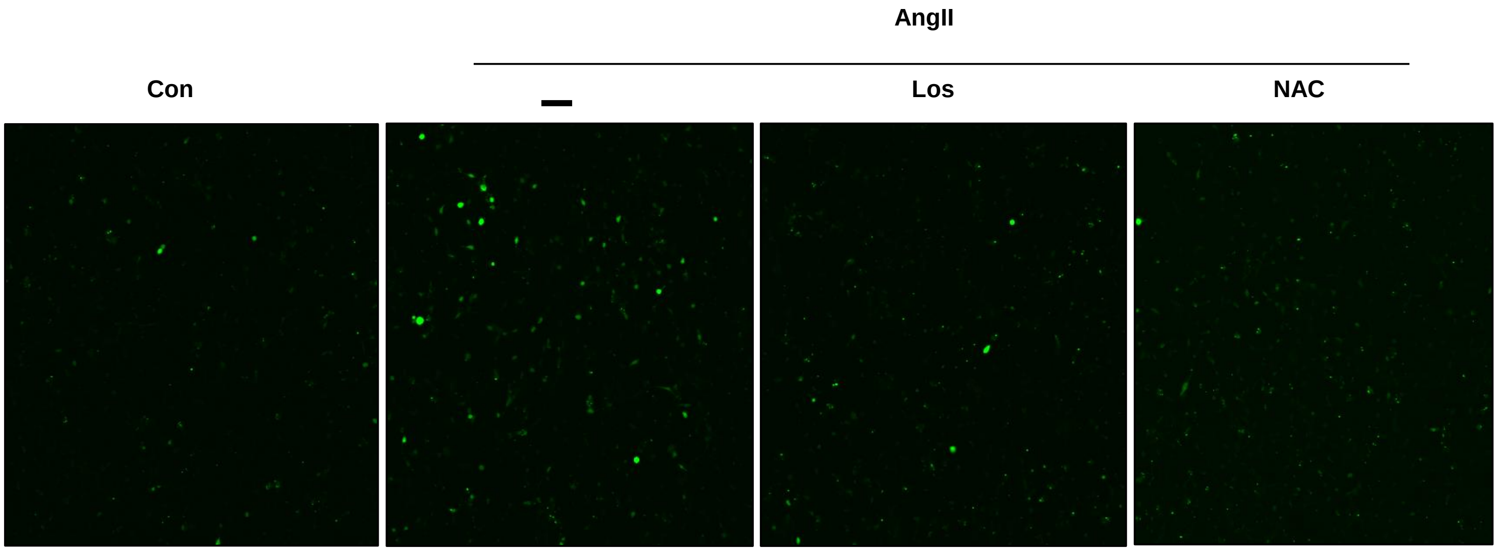

Supplement: Supplementary file 2 — unedited dataset 1 [file 41598_2019_44194_MOESM2_ESM.pdf]
